# Synthesis of New Promising BNCT Agents Based on Conjugates of *closo*-Dodecaborate Anion and Aliphatic Diamino Acids

**DOI:** 10.3390/ijms26010068

**Published:** 2024-12-25

**Authors:** Margarita N. Ryabchikova, Alexey V. Nelyubin, Ilya N. Klyukin, Nikita A. Selivanov, Alexander Yu. Bykov, Alexey S. Kubasov, Vsevolod A. Skribitsky, Yulia A. Finogenova, Kristina E. Shpakova, Anton A. Kasianov, Alexey A. Lipengolts, Andrey P. Zhdanov, Elena Yu. Grigoreva, Konstantin Yu. Zhizhin, Nikolay T. Kuznetsov

**Affiliations:** 1 Faculty of Chemistry, Higher School of Economics, Myasnitskaya St. 20, 101000 Moscow, Russia; ryabchikovaarita@gmail.com; 2 Kurnakov Institute of General and Inorganic Chemistry, Russian Academy of Sciences, Leninskii Pr. 31, 119991 Moscow, Russia; nelyubin.av@yandex.ru (A.V.N.); klukinil@igic.ras.ru (I.N.K.); goovee@yandex.ru (N.A.S.); bykov@igic.ras.ru (A.Y.B.); fobosax@mail.ru (A.S.K.); skvseva@yandex.ru (V.A.S.); shpakova.k.e@gmail.com (K.E.S.); lipengolts@mail.ru (A.A.L.); zhizhin@igic.ras.ru (K.Y.Z.); boron@igic.ras.ru (N.T.K.); 3 N.N. Blokhin National Medical Research Center of Oncology, 24, Kashirskoe Shosse, 115522 Moscow, Russia; b-f.finogenova@yandex.ru (Y.A.F.); a_kasianov@mail.ru (A.A.K.); grig-elen11@mail.ru (E.Y.G.); 4 Engineer and Physics Institute of Biomedicine, National Research Nuclear University “MEPhI”, 31, Kashirskoe Shosse, 115409 Moscow, Russia

**Keywords:** *closo*-dodecaborate, nitrilium derivatives, amino acids, BNCT, boron neutron capture therapy, toxicity, selective delivery, boronophenylalanine, BPA

## Abstract

In this work, a series of boronated amidines based on the *closo*-dodecaborate anion and amino acids containing an amino group in the side chain of the general formula [B_12_H_11_NHC(NH(CH_2_)_n_CH(NH_3_)COOH)CH_3_], where n = 2, 3, 4, were synthesized. These derivatives contain conserved α-amino and α-carboxyl groups recognized by the binding centers of the large neutral amino acid transporter (LAT) system, which serves as a target for the clinically applied BNCT agent para-boronophenylalanine (BPA). The paper describes several approaches to synthesizing the target compounds, their acute toxicity studies, and tumor uptake studies in vivo in two tumor models. The promising compound [B_12_H_11_NHC(NH(CH_2_)_2_CH(NH_3_)COOH)CH_3_]*3H_2_O demonstrates low toxicity (LD_50_ in a range from 150 to 300 mg/kg) and excellent solubility and also shows selective uptake in experimental melanoma in laboratory mice (T/N ratio remained >3 up to 60 min post-injection, with a maximum T/N of 6.2 ± 2.8 at 45 min).

## 1. Introduction

^10^Boron neutron capture therapy (BNCT) is an actively developing method for treating advanced cases of cancer. This method relies on the selective destruction of tumor cells that accumulate boron-containing agents upon irradiation with thermal neutrons. A distinguishing feature of BNCT compared to other radiation therapy methods is that the absorbed neutron dose is determined not only by the duration and intensity of the irradiation but also by the concentration of the ^10^B isotope in target cells. The effectiveness of BNCT largely depends on the selective accumulation and retention of the boron-10-containing agent within the tumor cells. BNCT agents must have low toxicity and the ability to selectively accumulate in tumor tissue, with a target-to-normal (T/N) tissue accumulation ratio of no less than 3:1, maintained for a duration sufficient to conduct therapeutic neutron irradiation [1].

In 2020, BNCT was approved in Japan by the National Health Insurance System as a routine treatment for malignant tumors, and currently, the only compound used is 4-dihydroxyboryl-L-phenylalanine (BPA) [2]. However, this compound has significant drawbacks, including low water solubility and a low boron content (less than 5% by mass). The only registered BNCT drug at present is Steboronin—a BPA-based compound complexed with sorbitol, administered through prolonged (up to 2-h) infusions, partially before irradiation and partially during irradiation [3].

Research for new agents is now mainly focused on increasing the delivered boron content by moving away from boronic acid derivatives and towards boron cluster derivatives and boron-based nanosystems [4,5,6,7]. BPA was the first compound shown to selectively deliver boron into tumor tissues. Early studies on the use of BPA demonstrated active transport mechanisms for boron carriers in tumor cells [8,9]. Further studies revealed that its main transporter is the large neutral amino acid transporter (LAT) system, predominantly LAT1 [10]. Investigations of LAT1-mediated uptake of various amino acids and their derivatives showed that the binding site recognizes the presence and relative positioning of conserved α-amino and α-carboxyl groups in the substrate molecule [11]. Furthermore, a large hydrophobic substituent on the side chain, typically substituted aromatic derivatives, plays a critical role, though its position and number complexly affect substrate uptake and elimination rates within cells.

The first boron cluster-containing amino acid derivative to meet LAT receptor uptake criteria was (o-carboranyl)-alanine, though it exhibited lower effectiveness than BPA [12]. Subsequent synthesis yielded a range of carborane amino acid derivatives with various spacer groups [13,14,15,16], including derivatives of unnatural cyclic amino acids, which showed promising results when used as boronic acid derivatives [17].

However, in vitro studies of carborane-containing amino acids highlighted issues such as low water solubility, requiring the introduction of hydrophilic groups, synthetic challenges in obtaining optically pure derivatives, and low stability in biological media [18,19]. This led to a shift towards using boron cluster anion derivatives, primarily the *closo*-dodecaborate anion. Due to the high chemical stability of these compounds, stringent reaction conditions are often required to introduce *exo*-polyhedral substituents. A solution to this issue involved modifying a preintroduced substituent, typically thiol-based.

A series of derivatives containing free amino and carboxyl groups were synthesized [20,21]. These derivatives demonstrated selectivity and absolute boron concentrations in tumor cells comparable to or slightly better than BPA [22,23]. The primary drawbacks of these derivatives include their complexity and the unbalanced double negative charge of the cluster, which increases the ionic strength of their solutions and may negatively impact intravenous administration.

**Figure 1:**
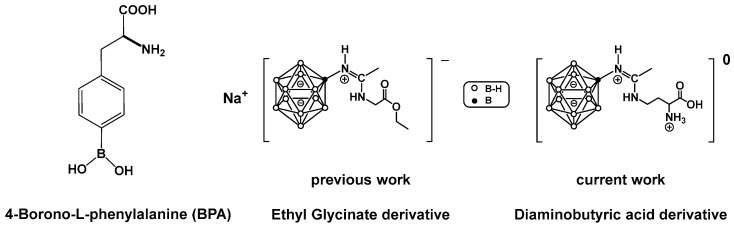
Structure of registered BNCT drug BPA and compounds studied in our research group in a melanoma model.

Another modification approach involves nucleophilic ring-opening of cyclic oxonium derivatives and conjugation with activated nitrilium derivatives. Using these methods, several amino acid derivatives were synthesized [24], and in the case of glycine derivatives, toxicity and in vivo distribution were studied (Figure 1). This study aims to synthesize conjugates of the *closo*-dodecaborate anion with diamino acids containing free amino and carboxyl groups and to investigate their biological properties.

## 2. Results

**Scheme 1:**
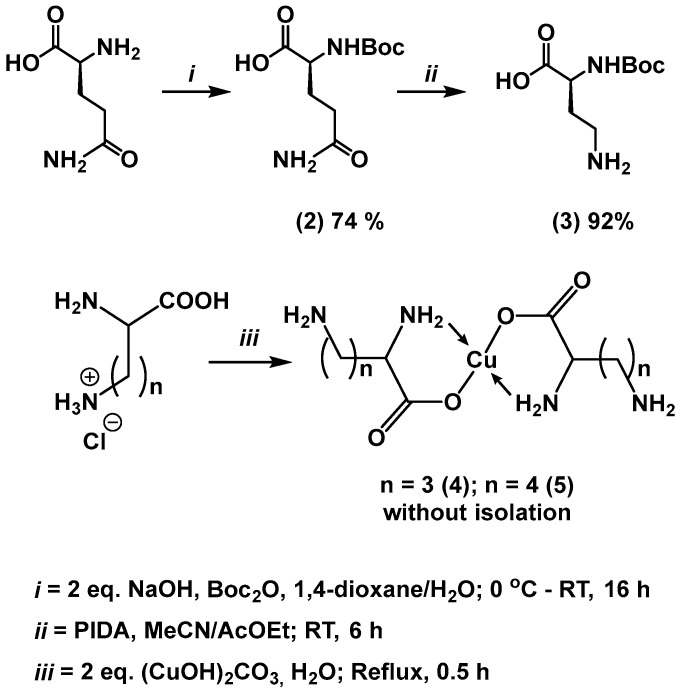
General scheme for the modification of diamino acids used in this work.

The activated *exo*-polyhedral nitrilium substituent in the nitrilium derivatives of the *closo*-dodecaborate anion can undergo nucleophilic addition reactions with various amines, alcohols, and thiols [25,26,27]. However, due to the slow reaction rate with O- and S-nucleophiles, derivatives containing a nucleophilic nitrogen atom were preferred for synthesizing amino acid derivatives with conserved α-amino and α-carboxyl groups. As starting substrates, we selected natural diamino acids with varying methylene spacer lengths (Scheme 1).

In the first stage, Nα-Boc-β-amino-L-alanine (**3**) was chosen as the amino acid component. This unnatural diamino acid can be synthesized from N-protected glutamine through a Hofmann-type reaction using a hypervalent iodine compound (phenyliodine (III) diacetate, PIDA) as the oxidant [28].

**Scheme 2:**
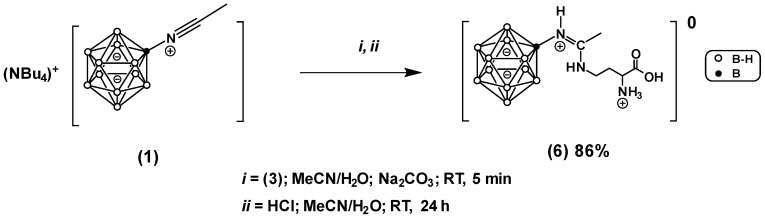
Synthesis scheme of a derivative based on γ-diamino butyric acid.

**Figure 2:**
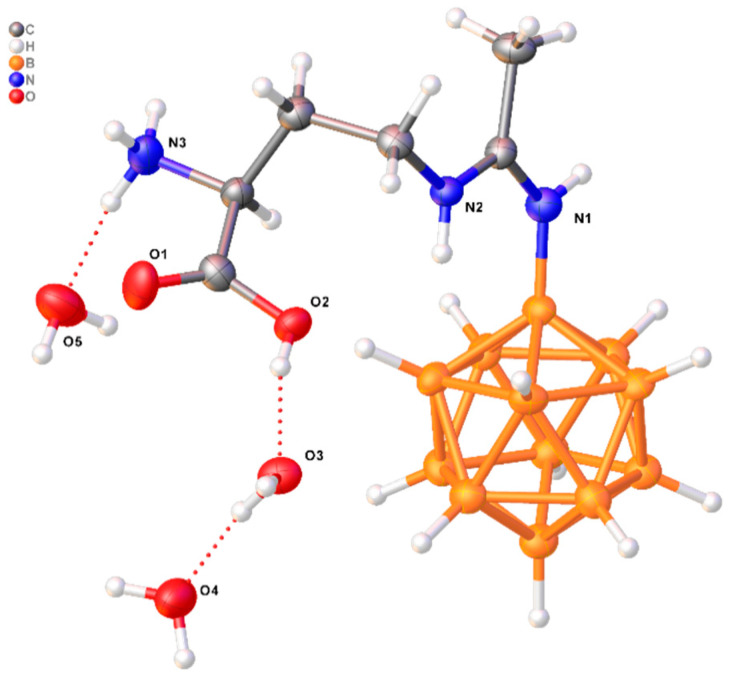
Structure of compound (**6**).

Since derivative (**3**) exists as a zwitterion, for deprotonation of the γ-amino group, the nucleophilic addition reaction to the nitrilium derivative (**1**) was conducted in an aqueous-organic medium (Scheme 2). It was determined that the highest yield, as measured by ^11^B NMR spectroscopy, was achieved using a mixture of acetonitrile/THF/saturated aqueous Na_2_CO_3_ in a 2:1:1 ratio. For biological studies, the N-tetrabutylammonium cation was replaced by a sodium ion through a reaction with sodium tetraphenylborate. After deblocking the α-amino group, the product precipitated as an internal salt of the form [B_12_H_11_NHC(NH(CH_2_)_2_CH(NH_3_)COOH)CH_3_]*3H_2_O (**6**). Recrystallization of the product from water yielded crystals suitable for structural analysis by X-ray diffraction (Figure 2), confirming the absence of sodium cations in the final derivative.

An additional experiment was conducted where the removal of the tert-butoxycarbonyl group was carried out without replacing the N-tetrabutylammonium cation. This modification required conducting the reaction in an aqueous-organic medium and produced a derivative (**6**) with an 86% yield.

Direct interaction of the acetonitrile derivative (**1**) with lysine predictably led to a mixture of three regioisomers, which could not be separated using chromatographic methods. This issue could not be resolved by simply adjusting the pH of the reaction medium and required the use of an α-amino-protected amino acid [29].

For natural amino acids (Orn, Lys), obtaining Nα-Boc derivatives requires a complex multistep synthesis involving the installation of a temporary orthogonal protective group on the side-chain amino group. A simpler method for deactivating the α-amino group involves complexation with copper (II) ions. Due to the chelating properties of α-amino acids and the high stability of square-planar Cu^2+^ complexes with a d9 electronic configuration, it is possible to protect the α-amino group, thereby enabling selective addition reactions at the side-chain amino group of diamino acids [30].

**Scheme 3:**
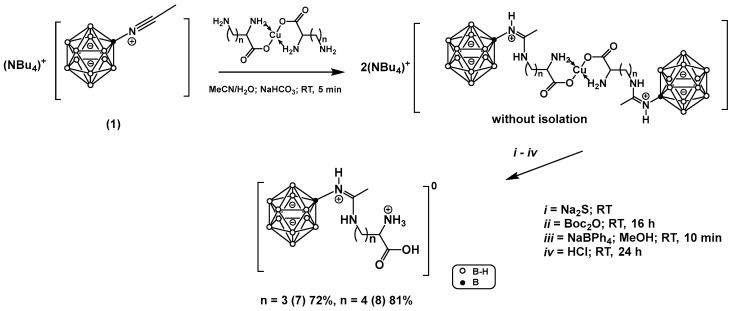
General scheme for the modification of diamino acids used in this work.

The interaction of compound (**1**) with copper complexes of ornithine and lysine (**4**) and (**5**) in an aqueous-acetonitrile solution, followed by the decomposition of the copper complex with sodium sulfide, resulted in N-tetrabutylammonium salts (**7**) and (**8**) respectively (Scheme 3). The yield of target boronated amidines, as determined by ^11^B NMR, was over 90%, with the main by-product being the corresponding boronated amide. Attempts to isolate these derivatives as internal salts, similar to derivative (**6**), were unsuccessful. Preparative flash chromatography on a reversed-phase HPLC column was proposed to isolate the target products. Using a water-acetonitrile mixture with trifluoroacetic acid as the eluent allowed for the isolation of the protonated forms (**7**) and (**8**) with yields of approximately 30%.

An attempt to replace the cation with sodium tetraphenylborate resulted in a reduced yield of 10–20% following chromatographic purification. This reduction was attributed to a side reaction during co-precipitation of the protonated form of the target derivative in an aqueous medium.

The final optimization step involved introducing Boc protection to the free amine fragment to prevent the formation of an α-ammonium cation that co-precipitates with tetrabutylammonium in the presence of sodium tetraphenylborate. After decomposing the copper complex, boron-containing derivatives were effectively separated from by-products via acetonitrile extraction. Treating derivatives (**7**) and (**8**) with Boc_2_O resulted in derivatives with Nα-Boc protection. Subsequent cation exchange with sodium proceeded similarly to derivative (**6**). Deblocking the amino group, followed by chromatographic purification, yielded the target derivatives (**7**) and (**8**) as internal salts with yields of 72% and 81%, respectively. Spectral data is provided in Supplementary Materials (Figures S1–S18).

The structures of compounds (**6**) and (**7**) were determined by single-crystal X-ray diffraction. Unfortunately, for compound (**8**), it was not possible to obtain single crystal samples suitable for analysis.

**Figure 3:**
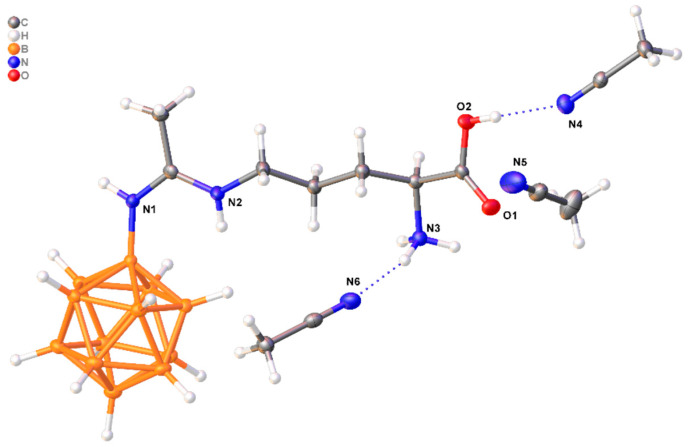
Structure of compound (**7**).

Compounds (**6**) and (**7**) crystallize in the monoclinic system (space group P2_1_). The crystallographically independent part of the compound (**6**) includes the zwitterionic compound B_12_H_11_NHC(Me)NHCH_2_CH_2_CH(NH_3_)COOH and three water molecules (Figure 2), while compound (**7**) consists of the zwitterionic B_12_H_11_NHC(Me)NHCH_2_CH_2_CH_2_CH(NH_3_)COOH and three CH_3_CN molecules (Figure 3). Solvent molecules are bound to the zwitterions in the crystals via strong OH…O(N) and NH…O(N) hydrogen bonds (Table 1 and Table 2), as well as weaker OH…HB and CH…HB contacts.

**Figure 4:**
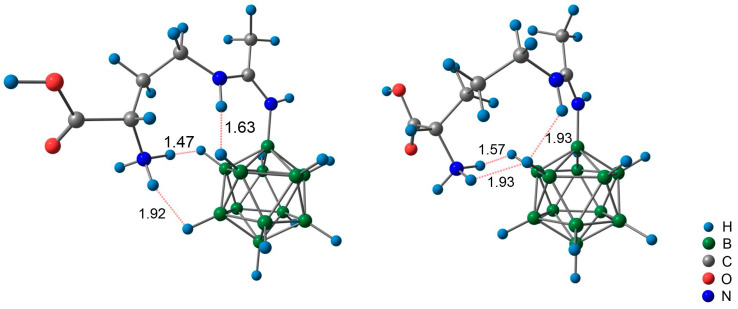
Optimized structures of systems (**6**) and (**7**).

In addition, the structures of (**6**) and (**7**) systems were analyzed with the help of quantum chemistry tools (Figure 4).

Firstly, the bond order of different types of CN bonds was estimated with the help of the Wiberg approach on natural atomic orbitals basis. In our previous research, it has been established that in the case of initial nitrilium derivatives of closo-dodecaborate anion [B_12_H_11_NCR]^–^ the formal multiplicity of CN bond was 3, and Wiberg bond orders lay in the interval of 2.62–2.66 [31]. In the systems under consideration, there are three types of CN bonds. The first one is the bond between the N_imine_ atom and the C atom of the amidine group NH=C(NH_2_), the second one is between the N_amide_ atom and C of the amidine group, and the third one is the bond between N_amine_ and α-C atom of aminoacid residue. A strong delocalization between CN_imine_ and CN_amide_ bonds was established. However, the CN_imine_ bond had the greatest values (1.50–1.52) of bond order compared to CN_amide_ (1.27–1.28). These results are very similar to the analogous amidine derivative of closo-dodecaborate anion [B_12_H_11_N=C(NH_2_)R]^–^. In the case of this derivative, the CN_imine_ bond has the 1.51 value of the Wiberg bond order, and the CN_amide_ bond is equal to 1.31. CN_amide_ bond of (**6**) and (**7**) structures due to partial delocalization with CN_imine_ bond has the greatest value of bond order compared to CN_amine_ (0.95–0.96).

The atomic charges of N_amide_ and N_imine_ were close to each other and lie in intervals of −0.57 to −0.61, which were lower than in the case of N_amine_ (−0.75). Carbonyl oxygen atoms had lower atomic charges (−0.56 to −0.57) compared to hydroxyl oxygen atoms (−0.65 to −0.66).

Non-covalent interactions play a crucial role in the stability and properties of *closo*-borate derivatives [25,26]. Analysis of non-covalent interactions was carried out with the help of QTAIM formalism. The geometry parameters of the optimized structure of (**6**) and (**7**) differed from analogous parameters in the crystal structure. The main reason for such phenomena is the prevailing of intermolecular contacts over intramolecular contacts in the crystal structure. In the crystal structures, dihydrogen bonds between NH_amide_ and BH groups were detected. For system (**7**), the length of such contacts is equal to 1.82 Å, and in the case of (**6**), the length is equal to 2.15 Å. The values of electron density at the bond critical point (bcp) are equal to 0.018 e Å^−3^ in the case of the anion (**7**) and 0.012 e Å^−3^ in the case of the anion (**6**). In structures obtained on the basis of geometry optimization in the gas phase, the number of noncovalent contacts between the boron cluster and the exo-polyhedral substituent is increased compared to crystal structures. In the gas phase of (**6**), NH_amide_ and BH contact is shortened and equal to 1.62 Å. The value of electron density is increased compared to crystal structure and is equal to 0.031 e Å^−3^. In addition to NH_amide_–BH contact, the interactions between hydrogens of the NH_amine_ group and BH were observed. One of them has a bond length, which is equal to 1.92 Å, and values of electron density at the corresponding bcp are equal to 0.017 e Å^−3^. The second bond length is equal to 1.46 Å, and the value of electron density is equal to 0.043 e Å^−3^. In the case of (**7**)**,** NH_amide_–BH contact is equal to 1.92 Å, and the value of electron density is equal to 0.018 e Å^−3^. As in the case of (**6**), there are two contacts between hydrogens of the NH_amine_ group and BH. The first of them has a bond length equal to 1.92 Å with the value of electron density equal to 0.016 e Å^−3^. The second one is shorter, and the bond length is equal to 1.56 Å, and the value of electron density is equal to 0.035 e Å^−3^.

### 2.1. Safety and Tolerability of Compounds in Laboratory Animals

The safety of compound (**6**) was evaluated after intravenous administration to laboratory mice at three dosages: 75, 150, and 300 mg/kg of body weight. The 75 mg/kg dosage (27.5 mg of boron/kg) was chosen to correspond to the boron dosage used in studies of BPA. Since no toxic effects were observed in mice at 75 mg/kg of compound (**6**), dosage levels were subsequently increased twofold and fourfold. At 75 mg/kg and 150 mg/kg, there were no cases of lethality or signs of acute toxicity (loss of consciousness, convulsion, or pain syndrome). However, at a dosage of 300 mg/kg, 100% lethality was observed. As LD50 value determination was out of the study scope, the dosage of 150 mg/kg was selected for subsequent tumor uptake studies as the maximum tolerated dose.

When compounds (**7**) and (**8**) were administered to laboratory mice at a dosage of 75 mg/kg, all animals in the group perished. Due to the high toxicity of these compounds, further studies were discontinued. Reducing the dosage was not considered, as it would have resulted in a boron dose lower than that achieved with BPA.

### 2.2. In Vivo Tumor Uptake

The in vivo tumor uptake of compound (**6**) was investigated in laboratory mice with two tumor models over a time range from 15 to 120 min following intravenous injection of 150 mg/kg of the compound.

**Figure 5:**
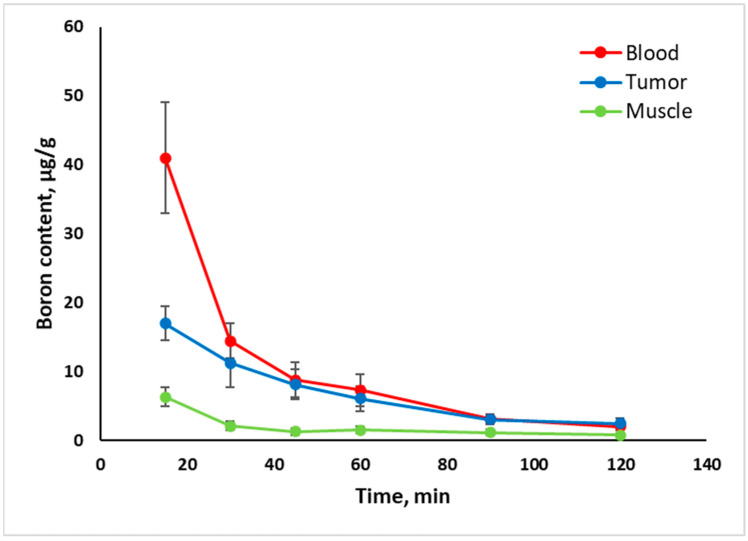
Dynamics of boron content in blood (red line), muscle tissue (green line), and B16F10 melanoma tumor tissue (blue line) following intravenous administration of compound (**6**) in C57Bl/6 mice.

The results of the compound (**6**) tumor uptake studies in C57Bl/6 mice with subcutaneous B16F10 melanoma are presented in Figure 5.

As expected, following intravenous administration, the maximum boron concentration in blood (41 ± 8 μg/g) was observed at the earliest time point, 15 min post-injection (Figure 5). Subsequently, boron levels in the blood decreased rapidly, reaching 9 ± 3 μg/g at 45 min and 2.1 ± 0.5 μg/g at 120 min post-injection (see Supplementary Table S2).

In muscle tissue, the maximum boron concentration (6.3 ± 1.4 μg/g) was only observed at the earliest time point due to high circulating boron levels. Thereafter, boron concentrations in muscle tissue remained within a range of 0.7–2.8 μg/g.

In tumor tissue, boron concentration reached a significant level of 17 ± 2 μg/g within 15 min. Although boron levels in the tumor subsequently declined, the decrease was slight, with boron concentrations remaining at 6.1 ± 1.8 μg/g even at 60 min post-injection.

**Figure 6:**
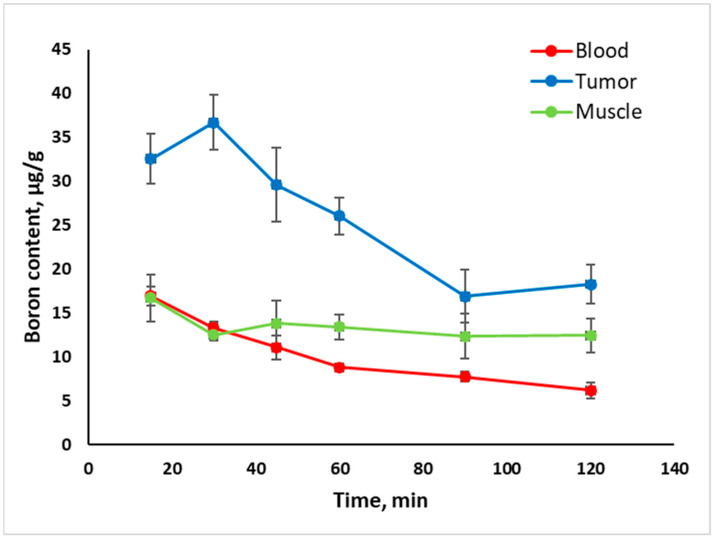
Dynamics of boron content in blood (red line), muscle tissue (green line), and B16F10 melanoma tumor tissue (blue line) following intravenous administration of BPA to C57Bl/6 laboratory mice.

As a reference compound, BPA, currently used in clinical practice, was studied (Figure 6). For tumor uptake studies, C57Bl/6 mice with subcutaneous B16F10 melanoma were administered 580 mg/kg of BPA, equivalent to 28 mg/kg of boron. Following intravenous injection, boron levels in the blood decreased slowly, from 17 ± 1 μg/g at 15 min post-injection to 6.2 ± 0.9 μg/g at 120 min. Boron accumulation in muscle tissue was substantial and remained steady throughout the observation period, measuring 17 ± 3 μg/g at 15 min and 12 ± 2 μg/g at 120 min post-injection (see Supplementary Table S3). Boron accumulation in tumor tissue was pronounced, with a maximum concentration of 37 ± 3 μg/g observed at 30 min, followed by a subsequent decrease.

**Figure 7:**
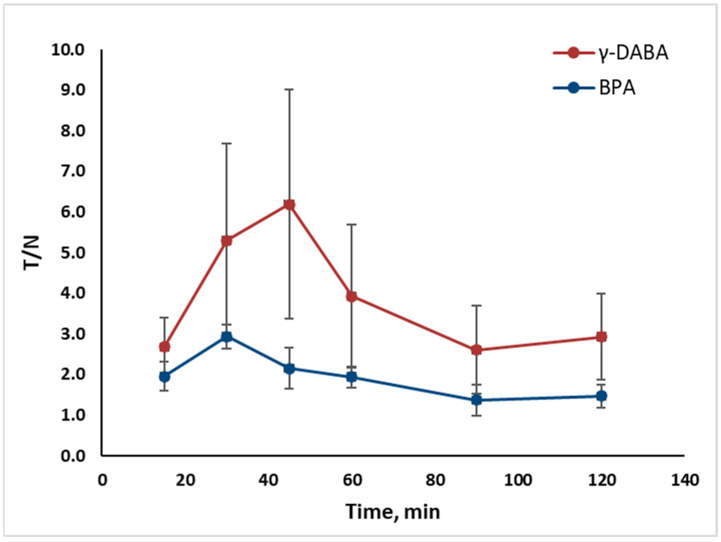
Boron content ratio in tumor and muscle tissue (T/N Ratio) following intravenous administration of compound (**6**) (red line) and BPA (blue line) to C57Bl/6 laboratory mice with B16F10 melanoma.

Since successful boron neutron capture therapy requires not only a high concentration of boron in the tumor but also a significant concentration gradient between the tumor and surrounding healthy tissue, we calculated the T/N ratio, which reflects the ratio of boron concentrations in the tumor to that in muscle tissue (Figure 7).

The slower clearance of compound (**6**) from tumor tissue resulted in a significantly higher boron concentration in the tumor compared to muscle tissue within the time range of 10 to 60 min. The maximum T/N ratio of 6.2 ± 2.8 was achieved 45 min after intravenous injection, followed by a decrease to 2.6 ± 1.1 at 90 min. In contrast, for BPA, a T/N ratio of 2.9 ± 0.3 was reached 30 min post-injection but did not persist even for 15 min. At all other time points within the 15–120-min range, the T/N ratio did not exceed 2.1 ± 0.5 (see Supplementary Table S4).

**Figure 8:**
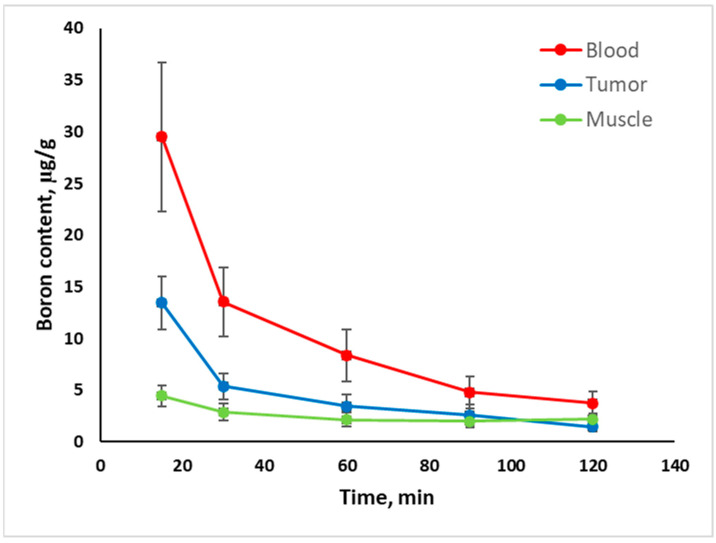
Dynamics of boron content in blood (red line), muscle tissue (green line), and 4T1 adenocarcinoma tumor tissue (blue line) following intravenous administration of compound (**6**) to Balb/C laboratory mice.

**Figure 9:**
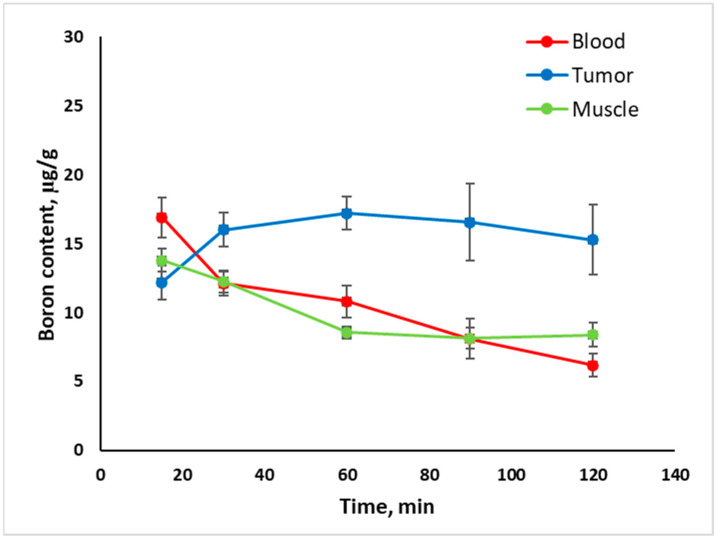
Dynamics of boron content in blood (red line), muscle tissue (green line), and 4T1 adenocarcinoma tumor tissue (blue line) following intravenous administration of BPA to Balb/C laboratory mice.

For subcutaneous 4T1 mammary carcinoma in Balb/C mice (Figure 8) similar to B16F10 melanoma, high boron concentration in blood (30 ± 7 μg/g) was observed 15 min after intravenous administration of the compound (**6**), followed by a rapid decrease. The boron concentration in muscle tissue did not exceed 2.9 ± 0.8 μg/g during the 30–120-min range. The maximum boron concentration in the 4T1 tumor was recorded at the earliest time point (13 ± 3 μg/g at 15 min post-injection) but then decreased quickly to no more than 5.4 ± 1.3 μg/g at 30 min. Thus, the dynamics of boron concentration change in 4T1 tumor tissue were characterized by significantly faster clearance compared to B16F10 melanoma. Conversely, BPA showed gradual accumulation in the 4T1 tumor, reaching a maximum concentration of 17 ± 1 μg/g at 60 min post-injection, with only a slight decrease to 15 ± 3 μg/g at 120 min (Figure 9, see Supplementary Tables S5 and S6).

**Figure 10:**
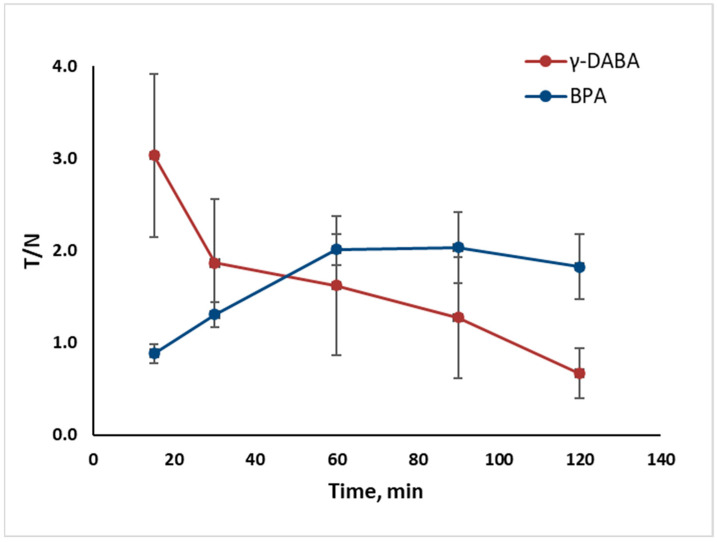
Boron content ratio in tumor and muscle tissue (T/N Ratio) following intravenous administration of compound (**6**) (red line) and BPA (blue line) to Balb/C laboratory mice with 4T1 adenocarcinoma.

The dynamics of the T/N ratio reflected the rapid clearance of compound (**6**) from 4T1 tumor tissue (Figure 10). The T/N ratio was 3.0 ± 0.9 at the first time point and subsequently dropped below 2, continuing to decrease throughout the observation period. In contrast, for BPA, the T/N ratio increased over 60 min, reflecting drug accumulation in the tumor, and then reached a plateau. It is noteworthy that 15 min post-injection of compound (**6**), the T/N ratio was 3.0 ± 0.9, whereas the maximum T/N for BPA was only 2.0 ± 0.4 (see Supplementary Table S7).

**Figure 11:**
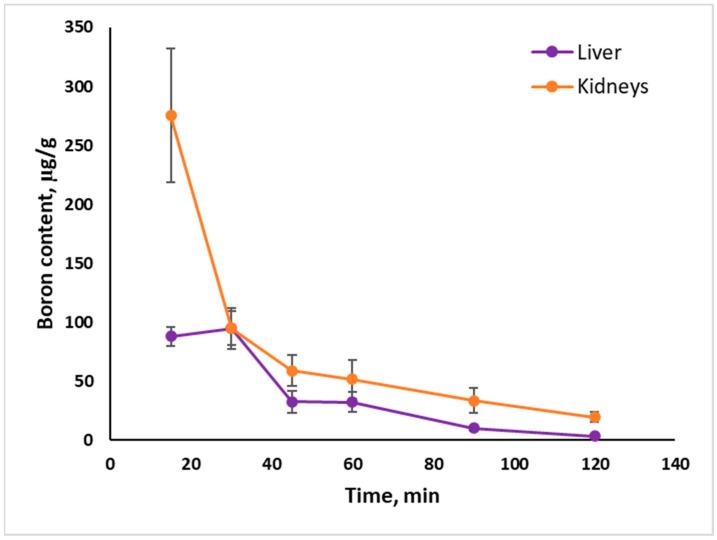
Dynamics of boron content in kidneys (orange line) and liver tissue (purple line) following intravenous administration of compound (**6**) in C57Bl/6 laboratory mice.

To evaluate possible excretion pathways for compound (**6**) in C57Bl/6 mice, excretory organs such as the kidneys and liver were also examined (Figure 11).

Boron concentration in the kidneys was initially high, measuring 275 ± 57 μg/g at 15 min post-injection, but then dropped rapidly to 95 ± 17 μg/g at 30 min and 20 ± 4 μg/g at 120 min. In contrast, boron levels in the liver were significantly lower, measuring 88 ± 8 μg/g at 15 min, decreasing to 33 ± 9 μg/g at 45 min, and continuing to decline. Since compound (**6**) is a low-molecular-weight compound, renal excretion appears to be the most likely pathway.

The dynamics of BPA accumulation in the kidneys and liver of C57Bl/6 mice were also studied. High initial boron concentrations were observed in both organs (202 ± 35 μg/g in the liver and 343 ± 19 μg/g in the kidneys), with a rapid decline to 29 ± 2 μg/g and 69 ± 6 μg/g in the liver and kidneys, respectively, within 30 min (see Supplementary Tables S2 and S3).

The promising compound (**6**) studied in this work demonstrated significantly higher solubility, enabling the administration of a boron dose twice that of BPA. Moreover, the low toxicity of compound (**6**) was confirmed by the absence of any toxic manifestations, even at twice the boron dose compared to boronophenylalanine.

An essential criterion for the suitability of a BNCT agent is the boron concentration ratio between tumor and healthy tissue. In this study, using the BPA as a reference, the maximum T/N ratio of 2.9 ± 0.3 for the B16F10 tumor model was achieved at 30 min post-injection, dropping below 2.6 within 15 min. These results align with the literature: T/N ~3 ratio is typical for BPA [32], while sodium borocaptate—a compound also used in clinical trials—may not even reach a T/N of 2 [33]. Compound (**6**) exhibited significantly more selective accumulation in melanoma tissue: throughout the 30–60-min interval following a single injection, the T/N ratio remained >3, with a maximum T/N of 6.2 ± 2.8 at 45 min. These findings suggest that it could potentially serve as the basis for a new BNCT agent suitable for single bolus administration.

It is noteworthy that in studies with 4T1 mammary adenocarcinoma, compound (**6**) did not demonstrate equally impressive results: the T/N ratio of 3.0 ± 0.9 achieved at 15 min post-injection fell below 2 thereafter, whereas BPA accumulation increased. These findings underscore the importance of selecting appropriate tumor models for preclinical studies and provide a rationale for expanding the panel of available agents to treat tumors of various localizations.

## 3. Materials and Methods

### 3.1. NMR Spectroscopy

^1^H, ^11^B, and ^13^C NMR spectra of the investigated compounds were recorded using a AVANCE-300 pulse Fourier spectrometer (Bruker, Ettlingen, Germany) at frequencies of 300.3, 96.32, and 75.49 MHz, respectively, with internal stabilization by deuterium. Tetramethylsilane or boron trifluoride etherate was used as external standards.

### 3.2. Analytical Reversed-Phase High-Performance Liquid Chromatography (RP-HPLC)

RP-HPLC analysis was performed on an isocratic Knauer HPLC system consisting of a PDA Smartline 2800 detector, Smartline 1000 pump, and a NanoChrome ChromCore 120 C18 column (250 × 4.6 mm, 5 µm particle size of the stationary phase). Sample injection was done manually, with a loop volume of 20 µL. Elution in a 35% MeCN/65% aqueous trifluoroacetic acid solution system with an acid content of 0.2% vol. To evaluate the purity of the derivatives, we used a method similar to that used for BPA [34]. Purity is accepted if the surface of the principal peak (at a wavelength of 210 nm.) is more than 98% of the integrated surface.

### 3.3. Preparative RP-HPLC

Preparative RP-HPLC was conducted on a Unique AutoPure100 FPLC chromatograph (Inscinstech, Suzhou, China) with a UV detector, an automatic fraction collector, and a 1000 µL loop. The separation was performed on a Hawach Spherical C18 Flash Column (SLC18SP10025PF) (Hawach Scientific, Xi’an, China). Fractions containing the target compound (volume of 12 mL) were automatically collected based on the UV detector signal at a wavelength of 232 nm. Eluent A was 99.8/0.2 H_2_O/CF_3_COOH, and Eluent B was 100% CH_3_CN, with a flow rate of 35 mL/min. Elution gradient: 0–10 min 15% A, 10–20 min 35% A, 20–30 min 35% A, and 30–40 min 90% A.

### 3.4. High-Resolution ESI Mass Spectrometry

High-resolution ESI mass spectra of compound solutions in acetonitrile were recorded using an LCMS-IT-TOF mass spectrometer (Shimadzu, Kyoto, Japan) in direct injection mode, with an m/z range of 120–700 Da. The detector voltage was set at 1.55 kV, and the ESI voltage at 4.50 kV. Equipment tuning (mass calibration and sensitivity check) was performed prior to analysis.

### 3.5. X-Ray Crystal Structure Determination

Single-crystal X-ray diffraction data for compounds 1 and 2 were collected on a three-circle D8 Venture diffractometer (T = 100 K, graphite monochromator, ω and φ scanning mode) (Bruker, Madison, WI, USA). Data were indexed and integrated using the SAINT program (version 8.40A) [35], then scaled and corrected for absorption using the SADABS program (version 2.03) [36]. Details are provided in Table S1.

The structures were determined by direct methods and refined using the full-matrix least-squares technique on F^2^ with anisotropic displacement parameters for non-hydrogen atoms. Hydrogen atoms were placed in calculated positions and refined within a riding model with fixed isotropic displacement parameters [U_iso_(H) = 1.5U_eq_(O), 1.5U_eq_(C) for CH_3_ groups, and 1.2U_eq_(C) for other groups]. Calculations were performed using the SHELXTL program (version 2014/7) [37] and the OLEX2 program package (version 1.5) [38].

Crystallographic data for all investigated compounds have been deposited with the Cambridge Crystallographic Data Center (CCDC) under accession numbers CCDC 2390349, 2390350. Copies of this data can be obtained free of charge from the CCDC, 12 Union Road, Cambridge CB2 1EZ, UK (Fax: +44 1223 336033; e-mail: deposit@ccdc.cam.ac.uk or via https://www.ccdc.cam.ac.uk (accessed on 19 December 2024)).

### 3.6. Sample Preparation and Determination of Boron in Biological Samples

Sample digestion was performed using microwave decomposition with an Ethos Easy system (MILESTONE, Sorisole BG, Italy). Biological tissue samples were placed in a fluoropolymer autoclave, followed by the addition of 5 mL of concentrated nitric acid (HNO_3_) and 1 mL of hydrogen peroxide (H_2_O_2_). The temperature of each autoclave was automatically controlled by an internal thermocouple in the microwave system. The decomposition protocol consisted of a linear gradual heating over 30 min to 190 °C, followed by a 10-min hold at 190 °C. After cooling the autoclave to 50 °C, it was removed from the microwave system and opened in a fume hood. The contents were transferred to boron-free plastic Falcon tubes (50 mL capacity). To each sample, 50 µL of the surfactant Triton X-100 was added, and the volume was adjusted to 10 mL with deionized water.

Quantitative boron determination in biological samples was conducted using inductively coupled plasma optical emission spectroscopy (ICP-OES). The measurements were performed on a high-resolution ICP-OES spectrometer (PlasmaQuant 9100 Series, Analytik Jena, Jena, Germany) using a standard multicalibration solution (ICP multi-element standard solution IV, Supelco Certipur, Darmstadt, Germany). For boron concentration measurements, two emission wavelengths, 249.773 nm and 249.678 nm, were used. Each sample was measured three times at each wavelength. The obtained signal intensities were compared with a calibration curve (correlation coefficient R^2^ > 0.9999; measurement range from 0.05 µg B/mL to 4 µg B/mL), and quantitative values of boron concentration in the sample were determined. The concentration of boron in biological tissue or organs was calculated based on the dilution factor of the sample.

### 3.7. Computational Details

The ORCA 5.0.4 program package [39] was used for density functional theory (DFT) calculations. The full geometry optimization of all model structures was carried out at the R2SCAN-3c level of theory. All molecular species considered had closed electron shells, and the spin-restricted approximation was applied. The tight criteria of SCF convergence (Tight SCF) were employed during the calculations. For a more accurate evaluation of the electronic structures of the systems under consideration, additional single-point calculations on ωB97X-D3/TZVPP level of theory with no resolution of identity approximation (“NORI” keyword) were carried out. Symmetry operations were not applied during the geometry optimization procedure. The Hessian matrices were calculated numerically for all model structures to prove the location of correct minima on potential energy surfaces. No imaginary frequencies were found in any of the cases. The natural bond orbital (NBO) method was employed using the NBO7 program package [40]. Topological analysis of the electron density distribution within the Quantum Theory of Atoms in Molecules (QTAIM) formalism, developed by Bader [41], was carried out using the Multiwfn program (version 3.8) [42]. The visualization of optimized structures was carried out with the help of the ChemCraft program (version 1.7) [43].

### 3.8. Reagents and Materials

The starting amino acids from Sigma Aldrich (St. Louis, MO, USA) were used without additional purification. Solvents with 99% purity were used without additional purification. Trifluoroacetic acid was distilled immediately before use. The acetonitrile derivative of the *closo*-dodecaborate anion (**1**) was synthesized according to the known method [31]. Active pharmaceutical ingredient Boronophenylalanine (BPA) was purchased from Interpharma Praha (Praha, Czech Republic). BPA-fructose complex was synthesized in our in-house radiochemistry laboratory at the N.N. Blokhin National Medical Research Center of Oncology.

#### 3.8.1. Nα-(tert-Butoxycarbonyl)-L-glutamine (Boc-L-glutamine) (**2**)

8 g (0.2 mol, 2 equiv.) of NaOH was dissolved in 100 mL of water, cooled to 5 °C, and mixed with 14.6 g (0.1 mol equiv.) of glutamine. To the resulting solution, 100 mL of dioxane and 25.2 mL (24 g, 0.11 mol, 1.1 equiv.) of di-tert-butyl dicarbonate were added. The solution was stirred for 16 h at room temperature and concentrated using a rotary evaporator to approximately 75 mL. 1M NaHSO_4_ solution was added to adjust the pH to 3, resulting in the formation of a white emulsion. The target product was extracted three times with 80 mL portions of ethyl acetate. The combined organic fraction was washed with a saturated salt solution, dried over sodium sulfate, and evaporated. The product was recrystallized from ethyl acetate. Yield: 18.2 g (74%). The spectra matched the literature data [28].

^1^H NMR (CDCl_3_, δ, ppm): 6.86 (bs, 2H, CONH_2_), 5.76 (d, *J* = 7.7 Hz, 1H, NH), 4.28 (q, 1H, CH), 2.39 (m, 2H, CH_2_), 2.25–1.95 (m, 2H, CH_2_), 1.44 (s, 9H, C(CH_3_)_3_).

#### 3.8.2. Nα-Boc-β-amino-L-alanine (**3**)

Synthesized using a known method [44]. 9.85 g (0.04 mol, 1 equiv.) of compound (2) was suspended in 120 mL of an ethyl acetate-acetonitrile mixture (1:1 *v*/*v*). 15.5 g (0.048 mol, 1.2 equiv.) of (diacetoxyiodo)benzene and 30 mL of water were added to the suspension. The mixture was stirred at room temperature until completion of the reaction, as confirmed by HPLC (approximately 6 h). The solution was concentrated, and 40 mL of ethyl acetate was added, followed by ultrasonication for 20 min. The precipitate was separated by centrifugation and recrystallized from methanol. Yield: 7.99 g (92%). The spectra matched the literature data [45].

^1^H NMR (D_2_O, δ, ppm): 3.97 (m, 1H, CH), 3.07 (t, *J* = 7.8 Hz, 2H, γ-CH_2_), 2.22–1.89 (m, 2H, β-CH_2_), 1.44 (s, 9H, C(CH_3_)_3_).

### 3.9. General Method for the Synthesis of Copper Complexes of Diamino Acids

#### 3.9.1. Cu(Orn*HCl)_2_ (**4**) and Cu(Lys*HCl)_2_ (**5**)

10 mmol of the amino acid hydrochloride was dissolved in 20 mL of water. To the resulting solution, 20 mmol of (CuOH)_2_CO_3_ was added. The solution was boiled for 30 min. After cooling to room temperature, the solution was filtered to remove excess copper carbonate and used further without isolation or purification.

#### 3.9.2. [B_12_H_11_NHC(NH(CH_2_)_2_CH(NH_3_)COOH)CH_3_]*3H_2_O (**6**)

275 mg (1.25 mmol, 1.25 equiv.) of compound (**3**) was dissolved in 10 mL of water, and 66 mg (0.625 mmol, 1.25 equiv.) of Na_2_CO_3_ was added. To the resulting clear solution, 0.424 g (1 mmol, 1 equiv.) of compound (**1**) in 10 mL of acetonitrile was added. The solution was stirred vigorously for 5 min. After completion of the reaction (as confirmed by ^11^B NMR), the reaction mixture was concentrated using a rotary evaporator. The residue was treated with 10 mL of 1M hydrochloric acid and extracted three times with 10 mL portions of dichloromethane. The organic phase was separated and evaporated using a rotary evaporator. The dry residue was dissolved in 10 mL of acetonitrile, and 5 mL of concentrated hydrochloric acid was added. The solution was stirred for 24 h, concentrated using a rotary evaporator, and the product was recrystallized from water. Yield: 304 mg (86%).

^11^B NMR (CD_3_CN, δ, ppm): −8.0 (s, 1B, B(1)), −16.6 (d, J_B–H = 128 Hz, 10B, B(2–11)), −17.8 (d, 1B, B(12)). ^1^H NMR (CD_3_CN, δ, ppm): 8.03 (br s, 1H, NH=C(NH)–CH_3_), 6.92 (br s, 3H, NH_3_–CH–COOH), 6.77 (br s, 1H, NH=C(NH_2_)–CH_3_), 4.03 (t, *J* = 7.0 Hz, 1H, NH_3_–CH–COOH), 3.48 (m, 2H, γ-CH_2_), 2.25–2.07 (m, 2H, β-CH_2_), 2.11 (s, 3H, NH=C(NH)–CH_3_), 2.0–0.0 (m, 10H, B_12_H_11_). ^13^C{H} NMR (CD_3_CN, δ, ppm): 169.5 (NH_3_–CH–COOH), 166.2 (NH=C(NH)–CH_3_), 51.6 (NH_3_–CH–COOH), 40.1 (γ-CH_2_), 30.7 (β-CH_2_), 18.9 (NH=C(NH)–CH_3_). MS(ESI) *m*/*z* = 300.3073 (found for C_6_H_24_B_12_N_3_O_2_); calcd. for {[A]-H^−^} 300.3058. RP-HPLC retention time 7.44 min λ_max_ = 228 nm. Purity analysis HPLC > 99% area.

### 3.10. General Method for the Synthesis of Boronated Diamino Acid Derivatives

To a 3 mL aqueous solution of the copper complex of the corresponding amino acid (0.75 mmol, 1.5 equiv.), 250 mg (1.5 mmol, 1.5 equiv.) of NaHCO_3_ dissolved in 2 mL of water was added. A solution of 424 mg (1 mmol, 1 equiv.) of compound (**1**) in 10 mL of acetonitrile was prepared. The copper complex solution was added to the solution of compound (**1**) and stirred for 5 min. After completion of the reaction (as confirmed by ^11^B NMR), 5 mL of a concentrated NaCl solution was added to the reaction mixture. The organic layer was separated, and the aqueous layer was washed twice with 10 mL portions of acetonitrile. The combined organic fractions were concentrated to a volume of approximately 10 mL, and 10 mL of water was added. A concentrated Na_2_S solution was added until copper sulfide precipitated completely. The precipitate was separated by centrifugation. To the resulting orange solution, 414 µL (2 mmol, 2 equiv.) of Boc_2_O was added, and the solution was stirred at room temperature for 16 h. After completion of the reaction, acetonitrile was removed using a rotary evaporator, and the aqueous layer was acidified to pH = 2 with 1M hydrochloric acid and extracted three times with 15 mL portions of dichloromethane. The combined organic layer was evaporated using a rotary evaporator and dissolved in 15 mL of methanol. A solution of 342 mg (1 mmol, 1 equiv.) of NaBPh_4_ in 50 mL of water was added. The precipitate was separated, the solution was evaporated using a rotary evaporator, and the product was purified by flash chromatography.

#### 3.10.1. [B_12_H_11_NHC(NH(CH_2_)_3_CH(NH_3_)COOH)CH_3_]*3H_2_O (**7**)

Yield: 266 mg (72%).

^11^B NMR (CD_3_CN, δ, ppm): −7.1 (s, 1B, B(1)), −16.0 (d, J_B–H = 129 Hz, 10B, B(2–11)), −17.1 (d, 1B, B(12)). ^1^H NMR (CD_3_CN, δ, ppm): 7.95 (br s, 1H, NH=C(NH)–CH_3_), 6.73 (br s, 3H, NH_3_–CH–COOH), 6.63 (br s, 1H, NH=C(NH_2_)–CH_3_), 4.04 (br s, 1H, NH_3_–CH–COOH), 3.30 (q, *J* = 7.0 Hz, 2H, δ-CH_2_), 2.11 (s, 3H, NH=C(NH)–CH_3_), 1.94 (m, 2H, β-CH_2_), 1.70 (m, 2H, γ-CH_2_), 2.0–0.0 (m, 10H, B_12_H_11_). ^13^C{H} NMR (CD_3_CN, δ, ppm): 170.0 (NH_3_–CH–COOH), 165.8 (NH=C(NH)–CH_3_), 54.0 (NH_3_–CH–COOH), 43.3 (δ-CH_2_), 27.5 (β-CH_2_), 25.9 (γ-CH_2_), 18.9 (NH=C(NH)–CH_3_). MS(ESI) *m*/*z* = 314.3239 (found for C_6_H_24_B_12_N_3_O_2_); calcd. for {[A]-H^−^} 314.3214. RP-HPLC retention time 8.32 min λ_max_ = 228 nm. Purity analysis HPLC > 99% area.

#### 3.10.2. [B_12_H_11_NHC(NH(CH_2_)_3_CH(NH_3_)COOH)CH_3_]*3H_2_O (**8**)

Yield: 310 mg (81%).

^11^B NMR (CD_3_CN, δ, ppm): −7.0 (s, 1B, B(1)), −15.9 (d, J_B–H = 129 Hz, 10B, B(2–11)), −17.2 (d, 1B, B(12)). ^1^H NMR (CD_3_CN, δ, ppm): 7.90 (br t, *J* = 5.8 Hz, 1H, NH=C(NH)–CH_3_), 6.85 (br s, 3H, NH_3_–CH–COOH), 6.58 (br s, 1H, NH=C(NH_2_)–CH_3_), 4.00 (m, 1H, NH_3_–CH–COOH), 3.28 (q, *J* = 6.5 Hz, 2H, ε-CH_2_), 2.08 (s, 3H, NH=C(NH)–CH_3_), 2.0–1.8 (m, 2H, β-CH_2_), 1.63 (m, 2H, δ-CH_2_), 1.49 (m, 2H, γ-CH_2_), 2.0–0.0 (m, 10H, B_12_H_11_). ^13^C{H} NMR (CD_3_CN, δ, ppm): 170.4 (NH_3_–CH–COOH), 165.8 (NH=C(NH)–CH_3_), 54.1 (NH_3_–CH–COOH), 43.6 (ε-CH_2_), 30.0 (β-CH_2_), 29.4 (δ-CH_2_), 22.6 (γ-CH_2_), 18.9 (NH=C(NH)–CH_3_). MS(ESI) *m*/*z* = 328.3398 (found for C_6_H_24_B_12_N_3_O_2_); calcd. for {[A]-H^−^} 328.3371. RP-HPLC retention time 9.93 min λ_max_ = 228 nm. Purity analysis HPLC > 98.5% area.

### 3.11. Animal Studies

#### 3.11.1. Preparation of Solutions of Compounds (**6**)–(**8**) for Biological Studies

51 mg of each compound (dry weight) was dissolved in 0.5 mL of 0.1M NaOH. Distilled water (1.5 mL) was added to the solution, and the pH was adjusted to 7.1 with 1M NaOH. The solution volume was adjusted to 3 mL with distilled water and filtered through a 0.22 µm syringe filter.

#### 3.11.2. Safety and Tolerability Studies

The tolerability of the synthesized compounds was evaluated in female Balb/C mice weighing 20–22 g. The compounds were administered intravenously. Compound (**6**) was administered at three dosages: 75, 150, and 300 mg/kg of body weight. Compounds (**7**) and (**8**) were administered at a dosage of 75 mg/kg of body weight (see Results and Discussion).

In tumor uptake studies, female C57Bl/6 mice with B16F10 melanoma and female Balb/C mice with 4T1 mammary adenocarcinoma were used. The animals were obtained from the breeding facility at the N.N. Blokhin National Medical Research Center of Oncology, and tumor strains were received from the Center’s cryobank. To create tumor models, mice were subcutaneously injected with a freshly prepared suspension of tumor cells at a 1:6 mass/volume ratio in Hank’s solution in a volume of 60 µL into the right hind leg tibial region.

#### 3.11.3. Tumor Uptake of Compounds

The dynamics of biodistribution in tumor-bearing mice were studied for compound (**6**) and BPA as a reference compound.

The test compounds were administered intravenously at the following doses: compound (**6**)—150 mg/kg (55 mg of boron/kg) and BPA—580 mg/kg (28 mg of boron/kg). At specific time intervals, animals were anesthetized by inhalation of 2.5% isoflurane air mixture, and blood samples were taken from the anterior limb venous sinus. Following euthanasia via isoflurane overdose, organs and tissues were collected, including the tumor, muscle tissue, liver, and kidneys. Subcutaneous tumors were fully excised. For muscle tissue samples, the thigh muscles of the left (healthy) hind leg were excised. Liver samples weighing 0.4 to 1.6 g were collected, and both kidneys were completely removed using a “dry” method, with vessels clamped beforehand. Mice were grouped in sets of five for each time point.

In the study of compound (**6**) in C57Bl/6 mice with B16F10 melanoma, organ and tissue samples were collected at 15, 30, 45, 60, 90, and 120 min.

In the study of compound (**6**) in Balb/C mice with 4T1 adenocarcinoma, organ and tissue samples were collected at 15, 30, 60, 90, and 120 min.

In the study of BPA in C57Bl/6 mice with B16F10 melanoma, organ and tissue samples were collected at 15, 30, 45, 60, 90, and 120 min.

In the study of BPA in Balb/C mice with 4T1 adenocarcinoma, organ and tissue samples were collected at 15, 30, 60, 90, and 120 min.

All biological tissue and organ samples were placed in boron-free plastic containers. Samples were weighed on laboratory precision scales. The samples in containers were stored in a freezer at −20 °C.

## 4. Conclusions

In this work, three new compounds were synthesized with the potential for the development of new agents for BNCT. Two of these compounds exhibited high toxicity and were thus deemed unsuitable for biomedical use at this stage. The promising compound (**6**) demonstrated not only low toxicity and excellent solubility but also showed selective accumulation in experimental melanoma in laboratory mice. Thus, compound (**6**) is recommended for further in vivo BNCT studies. Also, further in silico experiments are planned to confirm the hypothesis of LAT1 binding efficiency for *closo*-dodecaborate anion compounds with conserved amino and carboxyl groups.

## Figures and Tables

**Table 1:** Hydrogen bonds in structure (**6**).

D-H…A	d(D-H), Å	d(H-A), Å	d(D-A), Å	D-H-A, °
O2-H2...O3	0.84	1.78	2.594 (5)	163.1
N3-H3A...O5	0.91	1.83	2.735 (7)	170.3
N3-H3C...O3 ^1^	0.91	2.16	2.814 (6)	127.7
O5-H5B...O4 ^2^	0.87	1.94	2.802 (8)	169.6
O3-H3F...O1 ^3^	0.87	2.07	2.827 (5)	145.5
O3-H3G...O4	0.87	1.86	2.726 (6)	172.3

^1^ +X, 1 + Y, +Z; ^2^ −1 − X, 1/2 + Y, −1 − Z; ^3^ −X, −1/2 + Y, −1 − Z 1.

**Table 2:** Hydrogen bonds in structure (**7**).

D-H…A	d(D-H), Å	d(H-A), Å	d(D-A), Å	D-H-A, °
O2-H2…N4	0.84	1.89	2.721 (2)	170.1
N3-H3B…O1 ^1^	0.91	2.18	2.894 (2)	135.1
N3-H3B…N4 ^1^	0.91	2.53	3.235 (2)	135.1
N3-H3C…N6	0.91	2.14	3.024 (2)	164.4

^1^ −X, −1/2 + Y, 1 − Z.

## Data Availability

Data is contained within the article.

## Supplementary Materials

The following supporting information can be downloaded at https://www.mdpi.com/article/10.3390/ijms26010068/s1.
