# Supplementary material for: Synthesis of New Promising BNCT Agents Based on Conjugates of closo-Dodecaborate Anion and Aliphatic Diamino Acids
Source: Int J Mol Sci. 2024 Dec 25;26(1):68. doi: 10.3390/ijms26010068 (PMC11719580; doi:10.3390/ijms26010068)

checkCIF/PLATON report

Structure factors have been supplied for datablock(s) cu_24kub37_0m_a

THIS REPORT IS FOR GUIDANCE ONLY. IF USED AS PART OF A REVIEW PROCEDURE FOR PUBLICATION, IT SHOULD NOT REPLACE THE EXPERTISE OF AN EXPERIENCED CRYSTALLOGRAPHIC REFEREE.

No syntax errors found. CIF dictionary Interpreting this report

Datablock: cu_24kub37_0m_a

Bond precision:	C-C = 0.0070 A		Wavelength=1.54178
Cell:	a=8.7039(3)	b=7.4599(3)	c=15.0553(7)
	alpha=90	beta=103.102(3)	gamma=90
Temperature:	100 K		

	Calculated	Reported
Volume	952.10(7)	952.10(7)
Space group	P 21	P 1 21 1
Hall group	P 2yb	P 2yb
Moiety formula	C6 H25 B12 N3 O2, 3(H2 O)	C6 H25 B12 N3 O2, 3(H2 O)
Sum formula	C6 H31 B12 N3 O5	C6 H31 B12 N3 O5
Mr	355.06	355.06
Dx,g cm-3	1.238	1.238
Z	2	2
Mu (mm-1)	0.650	0.651
F000	376.0	376.0
F000’	377.03	
h,k,lmax	10,9,18	10,9,18
Nref	3812[2060]	3500
Tmin,Tmax	0.710,0.937	0.446,0.754
Tmin’	0.644	

Correction method= # Reported T Limits: Tmin=0.446 Tmax=0.754
AbsCorr = MULTI-SCAN

Data completeness= 1.70/0.92 Theta(max)= 72.913

R(reflections)= 0.0752(3180) wR2(reflections)= 0.2188(3500)

S = 1.133 Npar= 248

The following ALERTS were generated. Each ALERT has the format
test-name_ALERT_alert-type_alert-level.
Click on the hyperlinks for more details of the test.

Alert level C

```
PLAT340_ALERT_3_C Low Bond Precision on  C-C Bonds ...............      0.007 Ang.
PLAT415_ALERT_2_C Short Inter D-H..H-X       H2E      ..H4D      .       2.00 Ang.
                                  -1-x,1/2+y,-1-z  =      2_454 Check
PLAT767_ALERT_4_C INS Embedded LIST 6 Instruction Should be LIST 4     Please Check
PLAT790_ALERT_4_C Centre of Gravity not Within Unit Cell: Resd.  #          1 Note
              C6 H25 B12 N3 O2
PLAT911_ALERT_3_C Missing FCF Refl Between Thmin & STh/L=    0.600         38 Report
                1  1  0,   2  0  0,   4  8  0,   8  0  0,  -4  8  1,  -2  0  1,
               -1  1  1,   1  1  1,   2  2  1,   4  8  1,  -6  7  2,  -3  0  2,
               -2  0  2,  -2  1  2,  -1  0  2,  -1  1  2,   0  0  2,   0  1  2,
                1  0  2,  -9  3  3,  -2  0  3,   0  1  3,  -2  0  4,   6  6  4,
               -2  0  5,  -1  0  5,  -1  2  5, -10  0  6,  -3  0  6,  -1  1  6,
              -10  1  7,   8  2  7,   2  7  9,  -9  2 10,   2  5 12,   2  5 13,
               -8  0 14,  -7  2 15,
PLAT913_ALERT_3_C Missing # of Very Strong Reflections in FCF ....         14 Note
                1  1  0,   2  0  0,  -2  0  1,   1  1  1,   2  2  1,  -3  0  2,
               -1  1  2,   1  0  2,  -2  0  3,  -2  0  4,  -2  0  5,  -1  0  5,
               -1  2  5,  -1  1  6,
```

Alert level G

```
PLAT002_ALERT_2_G Number of Distance or Angle Restraints on AtSite          2 Note
PLAT007_ALERT_5_G Number of Unrefined Donor-H Atoms .............         12 Report
              H1     H2     H2A    H3A    H3B    H3C    H3F    H3G    H4C    H4D    H5B
              H5C
PLAT072_ALERT_2_G SHELXL First  Parameter in WGHT  Unusually Large       0.13 Report
PLAT172_ALERT_4_G The CIF-Embedded .res File Contains DFIX Records          2 Report
PLAT415_ALERT_2_G Short Inter D-H..H-X       H3B      ..H6       .       2.10 Ang.
                                          x,1+y,z  =      1_565 Check
PLAT415_ALERT_2_G Short Inter D-H..H-X       H4C      ..H12      .       1.97 Ang.
                                  -1-x,1/2+y,-1-z  =      2_454 Check
PLAT415_ALERT_2_G Short Inter D-H..H-X       H5C      ..H8       .       2.04 Ang.
                                  -1-x,1/2+y,-1-z  =      2_454 Check
PLAT790_ALERT_4_G Centre of Gravity not Within Unit Cell: Resd.  #          2 Note
              H2 O
PLAT790_ALERT_4_G Centre of Gravity not Within Unit Cell: Resd.  #          3 Note
              H2 O
PLAT790_ALERT_4_G Centre of Gravity not Within Unit Cell: Resd.  #          4 Note
              H2 O
PLAT791_ALERT_4_G Model has Chirality at C5         (Sohncke SpGr)          S Verify
PLAT860_ALERT_3_G Number of Least-Squares Restraints ............           3 Note
PLAT912_ALERT_4_G Missing # of FCF Reflections Above STh/L=  0.600         28 Note
PLAT969_ALERT_5_G The 'Henn et al.' R-Factor-gap value ..........       2.938 Note
              Predicted wR2: Based on SigI**2  7.45 or SHELX Weight 19.30
PLAT978_ALERT_2_G Number C-C Bonds with Positive Residual Density.          0 Info
PLAT992_ALERT_5_G Repd & Actual _reflns_number_gt Values Differ by          2 Check
```

0 **ALERT level A** = Most likely a serious problem - resolve or explain
0 **ALERT level B** = A potentially serious problem, consider carefully

```
 6 ALERT level C = Check. Ensure it is not caused by an omission or oversight
16 ALERT level G = General information/check it is not something unexpected

 0 ALERT type 1 CIF construction/syntax error, inconsistent or missing data
 7 ALERT type 2 Indicator that the structure model may be wrong or deficient
 4 ALERT type 3 Indicator that the structure quality may be low
 8 ALERT type 4 Improvement, methodology, query or suggestion
 3 ALERT type 5 Informative message, check
```

It is advisable to attempt to resolve as many as possible of the alerts in all categories. Often the minor alerts point to easily fixed oversights, errors and omissions in your CIF or refinement strategy, so attention to these fine details can be worthwhile. In order to resolve some of the more serious problems it may be necessary to carry out additional measurements or structure refinements. However, the purpose of your study may justify the reported deviations and the more serious of these should normally be commented upon in the discussion or experimental section of a paper or in the "special_details" fields of the CIF. checkCIF was carefully designed to identify outliers and unusual parameters, but every test has its limitations and alerts that are not important in a particular case may appear. Conversely, the absence of alerts does not guarantee there are no aspects of the results needing attention. It is up to the individual to critically assess their own results and, if necessary, seek expert advice.

Publication of your CIF in IUCr journals

A basic structural check has been run on your CIF. These basic checks will be run on all CIFs submitted for publication in IUCr journals (*Acta Crystallographica*, *Journal of Applied Crystallography*, *Journal of Synchrotron Radiation*); however, if you intend to submit to *Acta Crystallographica Section C* or *E* or *IUCrData*, you should make sure that full publication checks are run on the final version of your CIF prior to submission.

Publication of your CIF in other journals

Please refer to the *Notes for Authors* of the relevant journal for any special instructions relating to CIF submission.

PLATON version of 22/08/2024; check.def file version of 21/08/2024

Datablock cu_24kub37_0m_a - ellipsoid plot

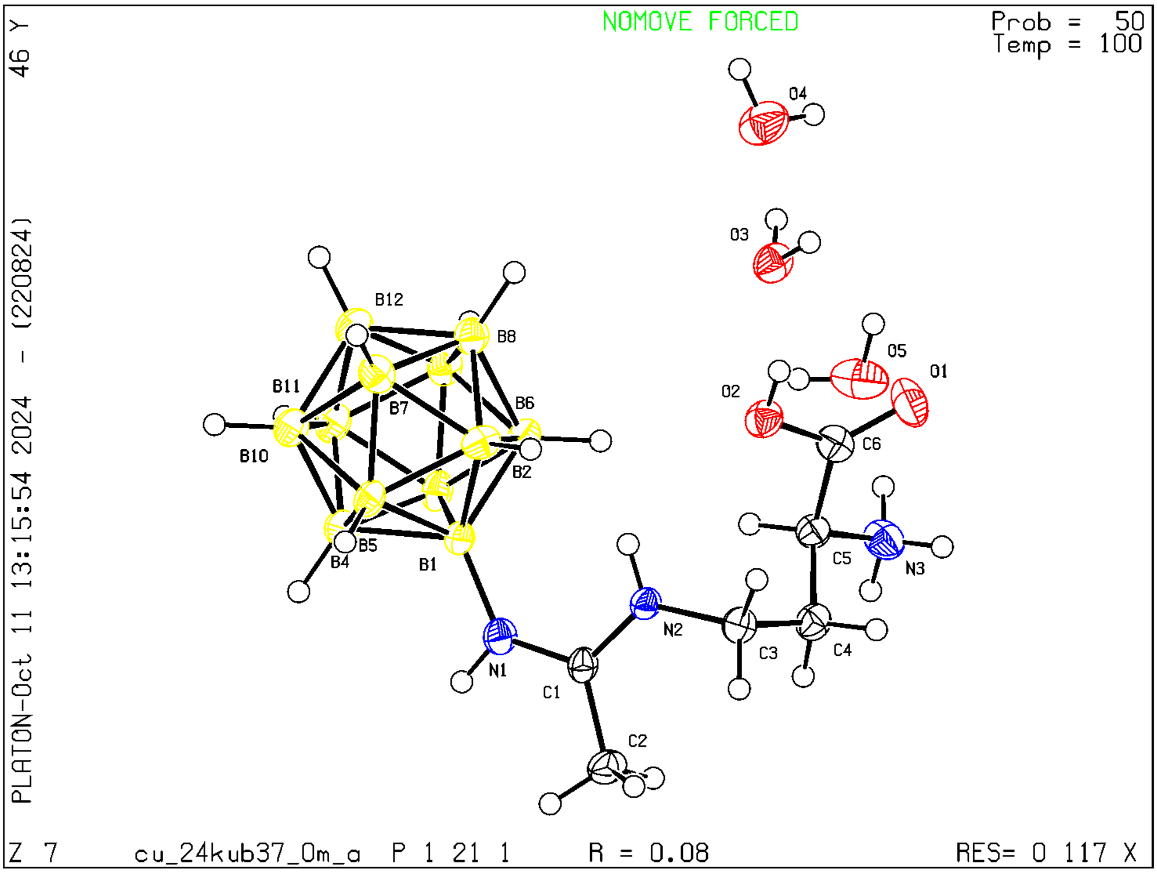

Supplement: Supplementary file 1 [file ijms-26-00068-s001.zip › 6_cifreport.pdf]
