# Supplementary material for: Synthesis of New Promising BNCT Agents Based on Conjugates of closo-Dodecaborate Anion and Aliphatic Diamino Acids
Source: Int J Mol Sci. 2024 Dec 25;26(1):68. doi: 10.3390/ijms26010068 (PMC11719580; doi:10.3390/ijms26010068)

# checkCIF/PLATON report

Structure factors have been supplied for datablock(s) 24kub221_0m_a

THIS REPORT IS FOR GUIDANCE ONLY. IF USED AS PART OF A REVIEW PROCEDURE FOR PUBLICATION, IT SHOULD NOT REPLACE THE EXPERTISE OF AN EXPERIENCED CRYSTALLOGRAPHIC REFEREE.

No syntax errors found. CIF dictionary Interpreting this report

## Datablock: 24kub221_0m_a

---

Bond precision: C-C = 0.0030 A Wavelength=0.71073

| | | | |
|---|---|---|---|
| Cell: | a=10.1504(6) | b=9.8851(6) | c=12.5694(8) |
| | alpha=90 | beta=91.566(2) | gamma=90 |
| Temperature: | 100 K | | |

| | Calculated | Reported |
|---|---|---|
| Volume | 1260.71(13) | 1260.71(13) |
| Space group | P 21 | P 1 21 1 |
| Hall group | P 2yb | P 2yb |
| Moiety formula | C7 H27 B12 N3 O2, 3(C2 H3 N) | C7 H27 B12 N3 O2, 3(C2 H3 N) |
| Sum formula | C13 H36 B12 N6 O2 | C13 H36 B12 N6 O2 |
| Mr | 438.20 | 438.20 |
| Dx,g cm-3 | 1.154 | 1.154 |
| Z | 2 | 2 |
| Mu (mm-1) | 0.069 | 0.069 |
| F000 | 464.0 | 464.0 |
| F000' | 464.11 | |
| h,k,lmax | 12,12,15 | 12,12,15 |
| Nref | 4982[ 2644] | 4962 |
| Tmin,Tmax | 0.979,0.982 | 0.652,0.746 |
| Tmin' | 0.978 | |

Correction method= # Reported T Limits: Tmin=0.652 Tmax=0.746
AbsCorr = MULTI-SCAN

Data completeness= 1.88/1.00 Theta(max)= 25.993

R(reflections)= 0.0316( 4720) wR2(reflections)= 0.0806( 4962)

S = 1.057 Npar= 304

The following ALERTS were generated. Each ALERT has the format
**test-name_ALERT_alert-type_alert-level.**
Click on the hyperlinks for more details of the test.

### Alert level C

```
STRVA01_ALERT_4_C           Flack parameter is too small
           From the CIF: _refine_ls_abs_structure_Flack   -0.800
           From the CIF: _refine_ls_abs_structure_Flack_su    0.400
PLAT244_ALERT_4_C Low    'Solvent' Ueq as Compared to Neighbors of        C10 Check
PLAT911_ALERT_3_C Missing FCF Refl Between Thmin & STh/L=    0.600          3 Report
                1   0   1,   -2   0 11,    4   0 13,
```

### Alert level G

```
PLAT007_ALERT_5_G Number of Unrefined Donor-H Atoms .............           6 Report
              H1     H2     H2A    H3A    H3B    H3C
PLAT032_ALERT_4_G Std. Uncertainty on Flack Parameter Value High .      0.400 Report
PLAT414_ALERT_2_G Short Intra D-H..H-X       H2A       ..H5        .      1.82 Ang.
                                                  x,y,z  =      1_555 Check
PLAT415_ALERT_2_G Short Inter D-H..H-X       H1        ..H6A       .      2.01 Ang.
                                             2-x,-1/2+y,2-z  =      2_747 Check
PLAT791_ALERT_4_G Model has Chirality at C6         (Sohncke SpGr)          S Verify
PLAT883_ALERT_1_G No Info/Value for _atom_sites_solution_primary .     Please Do !
PLAT933_ALERT_2_G Number of HKL-OMIT Records in Embedded .res File          1 Note
                1   0   1,
PLAT967_ALERT_5_G Note: Two-Theta Cutoff Value in Embedded .res ..       52.0 Degree
PLAT969_ALERT_5_G The 'Henn et al.' R-Factor-gap value ..........      1.797 Note
              Predicted wR2: Based on SigI**2  4.49 or SHELX Weight  7.63
PLAT978_ALERT_2_G Number C-C Bonds with Positive Residual Density.          5 Info
```

0 **ALERT level A** = Most likely a serious problem - resolve or explain
0 **ALERT level B** = A potentially serious problem, consider carefully
3 **ALERT level C** = Check. Ensure it is not caused by an omission or oversight
10 **ALERT level G** = General information/check it is not something unexpected

1 ALERT type 1 CIF construction/syntax error, inconsistent or missing data
4 ALERT type 2 Indicator that the structure model may be wrong or deficient
1 ALERT type 3 Indicator that the structure quality may be low
4 ALERT type 4 Improvement, methodology, query or suggestion
3 ALERT type 5 Informative message, check

It is advisable to attempt to resolve as many as possible of the alerts in all categories. Often the minor alerts point to easily fixed oversights, errors and omissions in your CIF or refinement strategy, so attention to these fine details can be worthwhile. In order to resolve some of the more serious problems it may be necessary to carry out additional measurements or structure refinements. However, the purpose of your study may justify the reported deviations and the more serious of these should normally be commented upon in the discussion or experimental section of a paper or in the "special_details" fields of the CIF. checkCIF was carefully designed to identify outliers and unusual parameters, but every test has its limitations and alerts that are not important in a particular case may appear. Conversely, the absence of alerts does not guarantee there are no aspects of the results needing attention. It is up to the individual to critically assess their own results and, if necessary, seek expert advice.

**Publication of your CIF in IUCr journals**

A basic structural check has been run on your CIF. These basic checks will be run on all CIFs submitted for publication in IUCr journals (*Acta Crystallographica*, *Journal of Applied Crystallography*, *Journal of Synchrotron Radiation*); however, if you intend to submit to *Acta Crystallographica Section C* or *E* or *IUCrData*, you should make sure that full publication checks are run on the final version of your CIF prior to submission.

**Publication of your CIF in other journals**

Please refer to the *Notes for Authors* of the relevant journal for any special instructions relating to CIF submission.

---

**PLATON version of 22/08/2024; check.def file version of 21/08/2024**

**Datablock 24kub221_0m_a** - ellipsoid plot

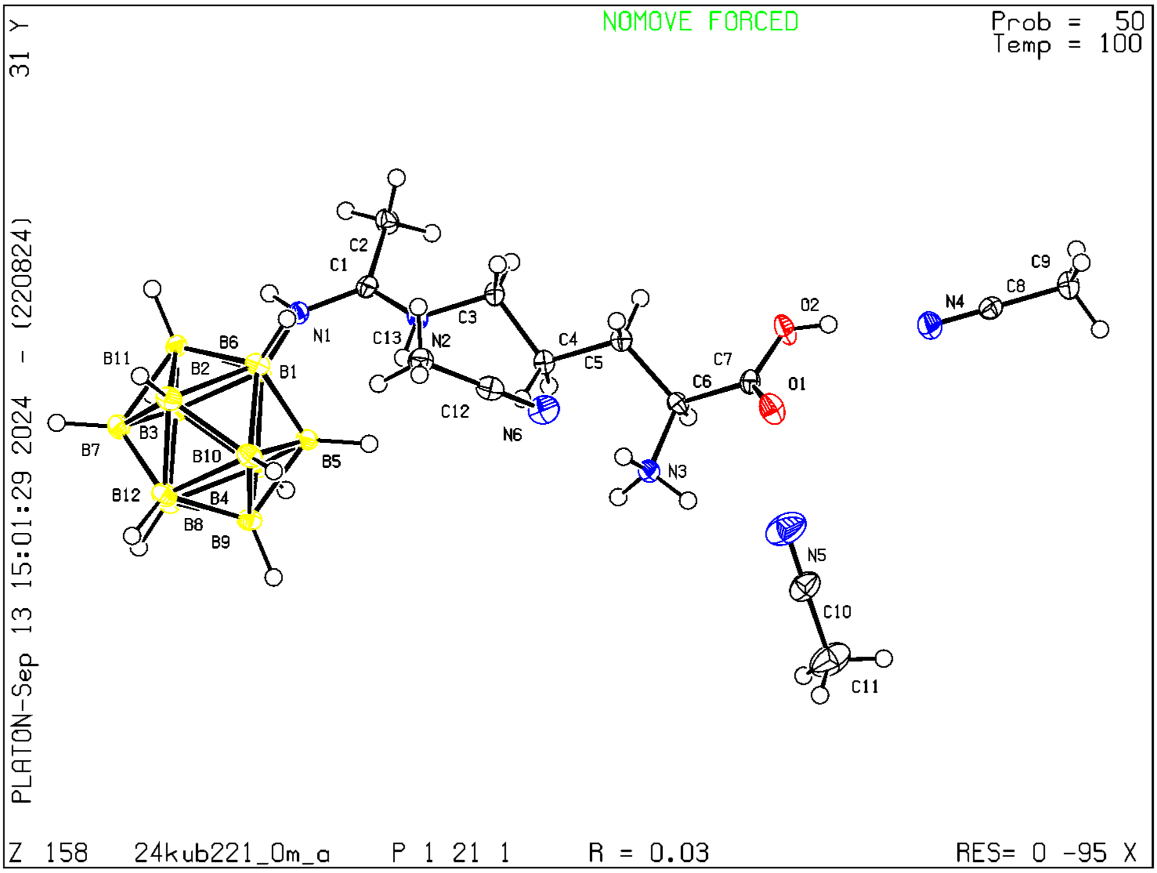

Supplement: Supplementary file 1 [file ijms-26-00068-s001.zip › 7_cifreport.pdf]
