# Supplementary material for: Synthesis of New Promising BNCT Agents Based on Conjugates of closo-Dodecaborate Anion and Aliphatic Diamino Acids
Source: Int J Mol Sci. 2024 Dec 25;26(1):68. doi: 10.3390/ijms26010068 (PMC11719580; doi:10.3390/ijms26010068)
Supplement: Supplementary file 1 [file ijms-26-00068-s001.zip › Ryabchikova_IJMS_suppl_mat.pdf]

Supporting Information for

Synthesis of New Promising BNCT Agents Based on Conjugates of *closo*-Dodecaborate Anion and Aliphatic Diamino Acids

Margarita N. Ryabchikova [1], Alexey V. Nelyubin [2], Ilya N. Klyukin [2], Nikita A. Selivanov [2], Alexander Yu. Bykov [2], Alexey S. Kubasov [2], Vsevolod A Skribitsky [2,3,4], Yulia A. Finogenova [3], Kristina E. Shpakova [2,3,4], Anton A. Kasyanov [3], Alexey A. Lipengolts [2,3,4], Andrey P. Zhdanov [2], Elena Yu. Grigoreva [3], Konstantin Yu. Zhizhin [2], and Nikolay T. Kuznetsov [2]

[1] Faculty of Chemistry, Higher School of Economics, Myasnitskaya St. 20, 101000, Moscow, Russia; ryabchikovaarita@gmail.com (M.N.R.)

[2] Kurnakov Institute of General and Inorganic Chemistry, Russian Academy of Sciences, Leninskii pr. 31, 119991 Moscow, Russia; nelyubin.av@yandex.ru (A.V.N.); klukinil@igic.ras.ru (I.N.K.); goovee@yandex.ru (N.A.S.); bykov@igic.ras.ru (A.Y.B.); fobosax@mail.ru (A.S.K.); zhdanov@igic.ras.ru (A.P.Z.); zhizhin@igic.ras.ru (K.Y.Z.); boron@igic.ras.ru (N.T.K.)

[3] N.N. Blokhin National Medical Research Center of Oncology; 24, Kashirskoe shosse, 115522, Moscow, Russia; skvseva@yandex.ru (V.A.S.); b-f.finogenova@yandex.ru (Y.A.F.); shpakova.k.e@gmail.com (K.E.S.); lipengolts@mail.ru (A.A.L.); grig-elen11@mail.ru (E.Y.G.)

[4] National Research Nuclear University "MEPhI", 31, Kashirskoe shosse, 115409, Moscow, Russia; a_kasianov@mail.ru (A.A.K.)

* Correspondence: zhdanov@igic.ras.ru;

Figure S1. $^{11}B\{^1H\}$ **NMR spectrum of** $[B_{12}H_{11}NHC(NH(CH_2)_2CH(NH_3)COOH)CH_3]*3H_2O$ **(6)**

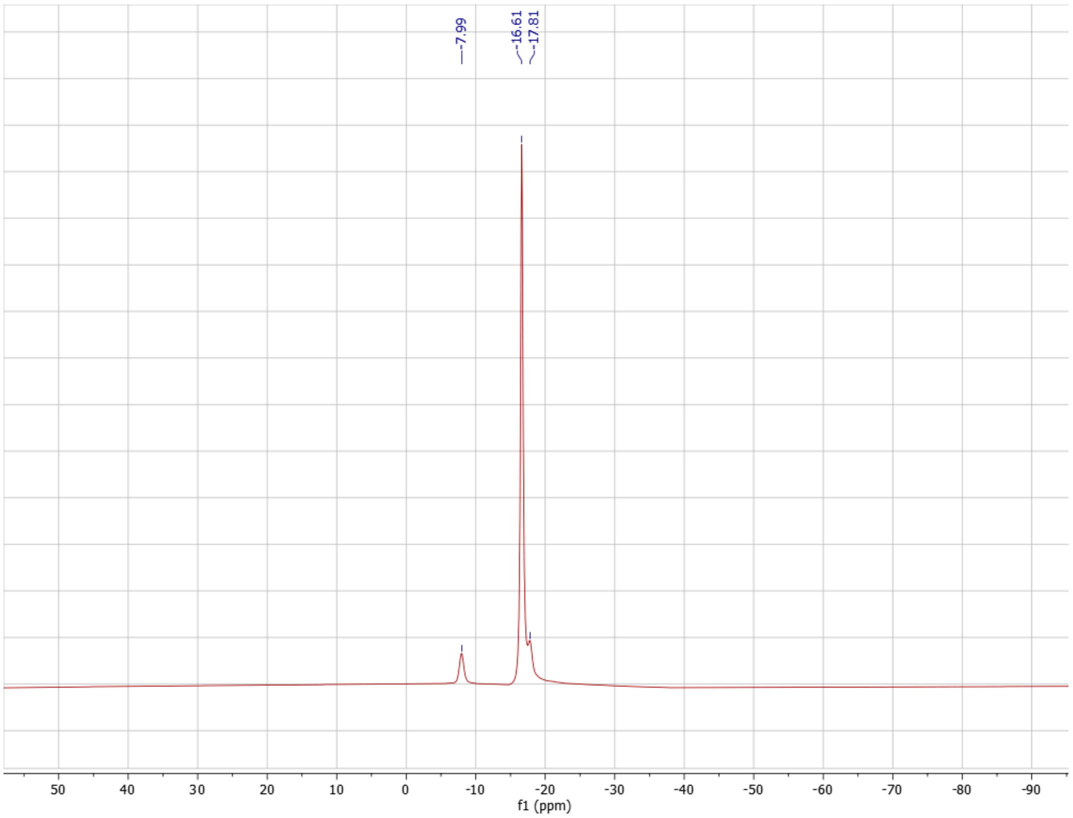

Figure S2. ^{11}B **NMR spectrum of** $[B_{12}H_{11}NHC(NH(CH_2)_2CH(NH_3)COOH)CH_3]*3H_2O$ **(6)**

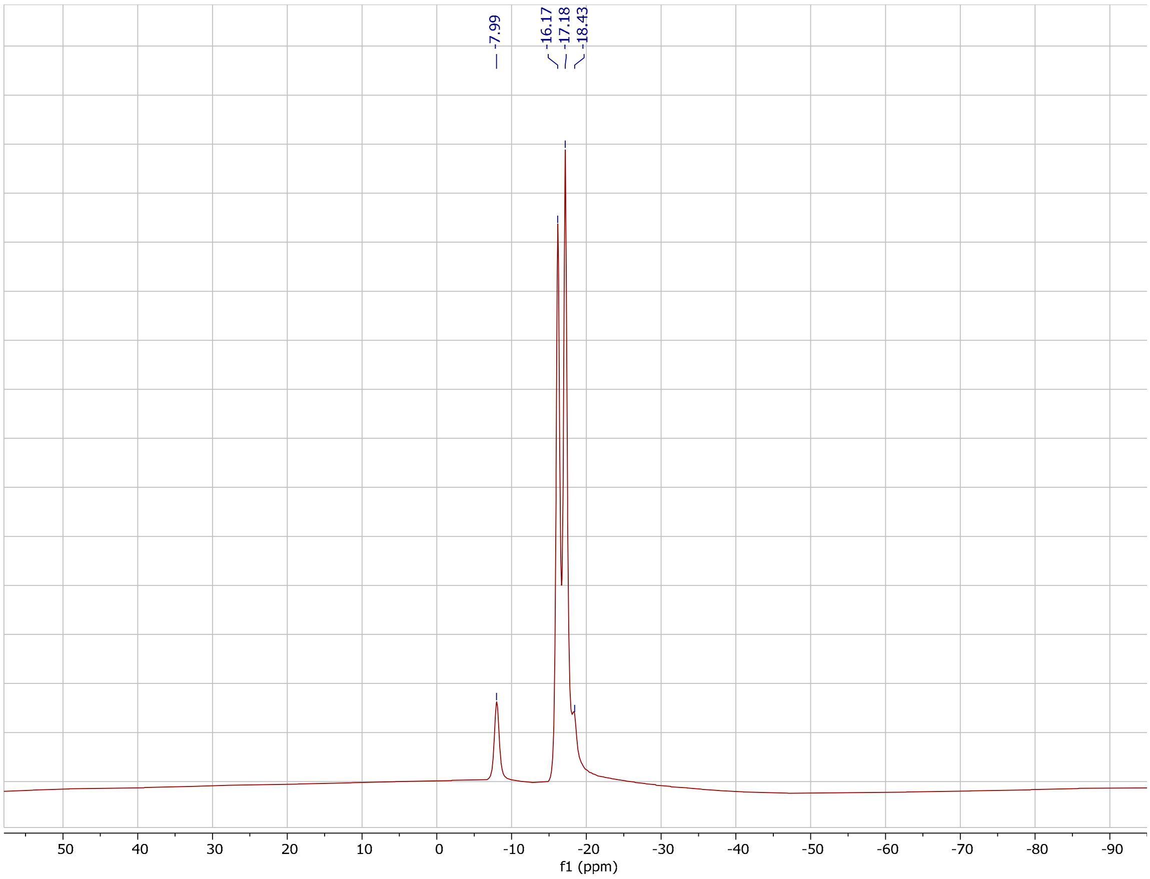

Figure S3. ^{1}**H NMR spectrum of [**$B_{12}H_{11}$**NHC(NH(**CH_2**)**$_2$**CH(**NH_3**)COOH)**CH_3**]*3**H_2O **(6)**

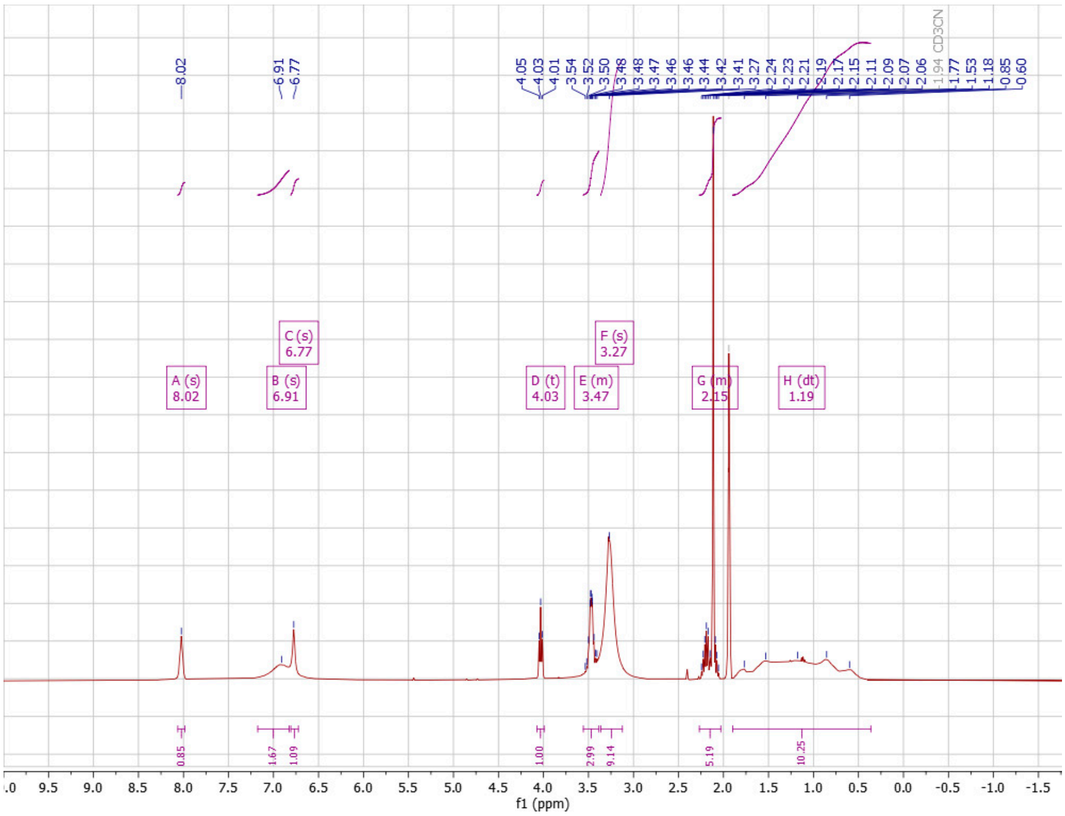

Figure S4. ^{13}**C{**^{1}**H} NMR spectrum of [**$B_{12}H_{11}$**NHC(NH(**CH_2**)**$_2$**CH(**NH_3**)COOH)**CH_3**]*3**H_2O **(6)**

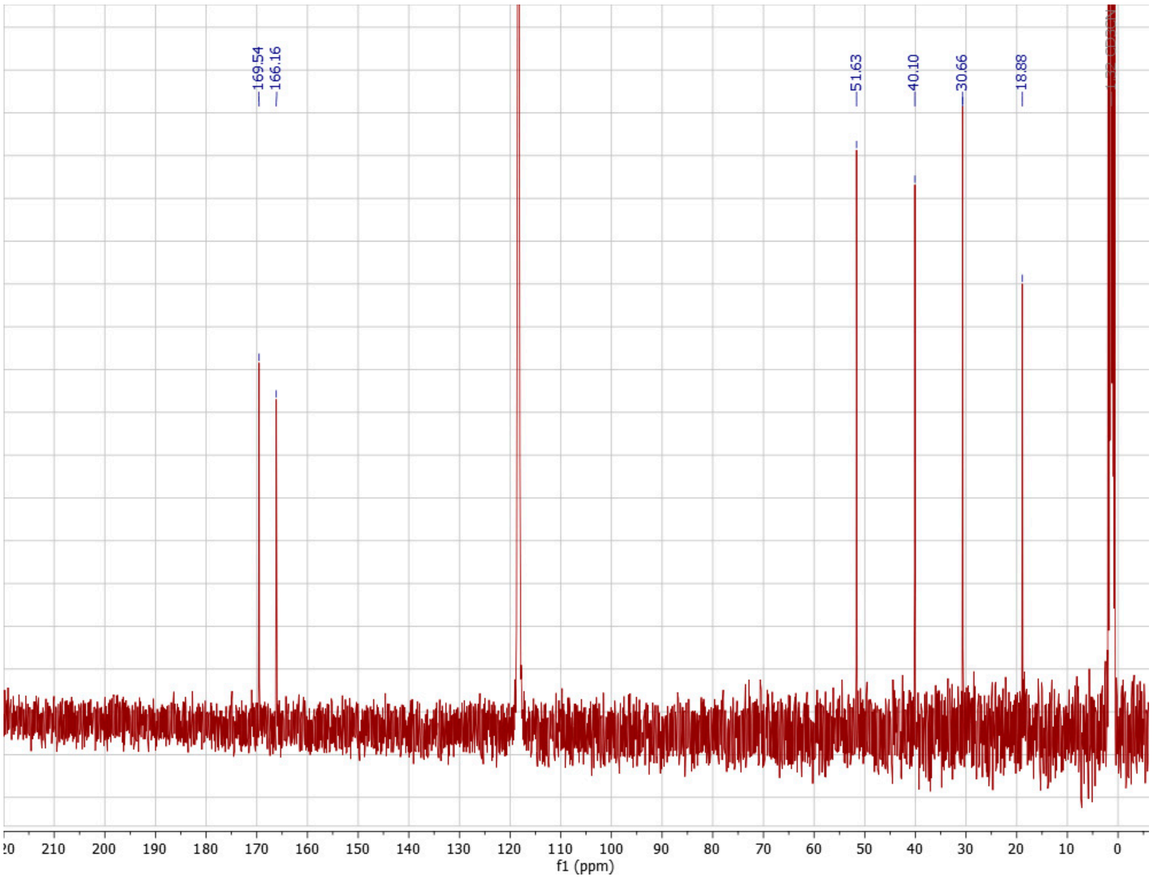

Figure S5. ESI-MS spectrum (anionic area) of $[B_{12}H_{11}NHC(NH(CH_2)_2CH(NH_3)COOH)CH_3]*3H_2O$ (6)

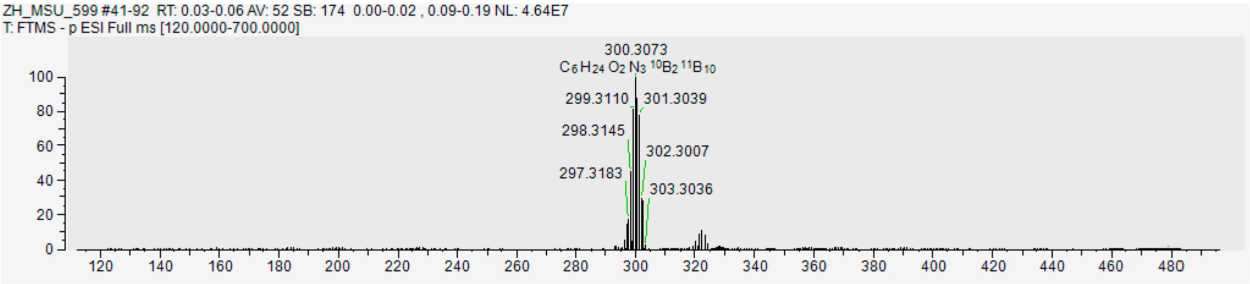

Figure S6. HPLC trace of $[B_{12}H_{11}NHC(NH(CH_2)_2CH(NH_3)COOH)CH_3]*3H_2O$ (6)

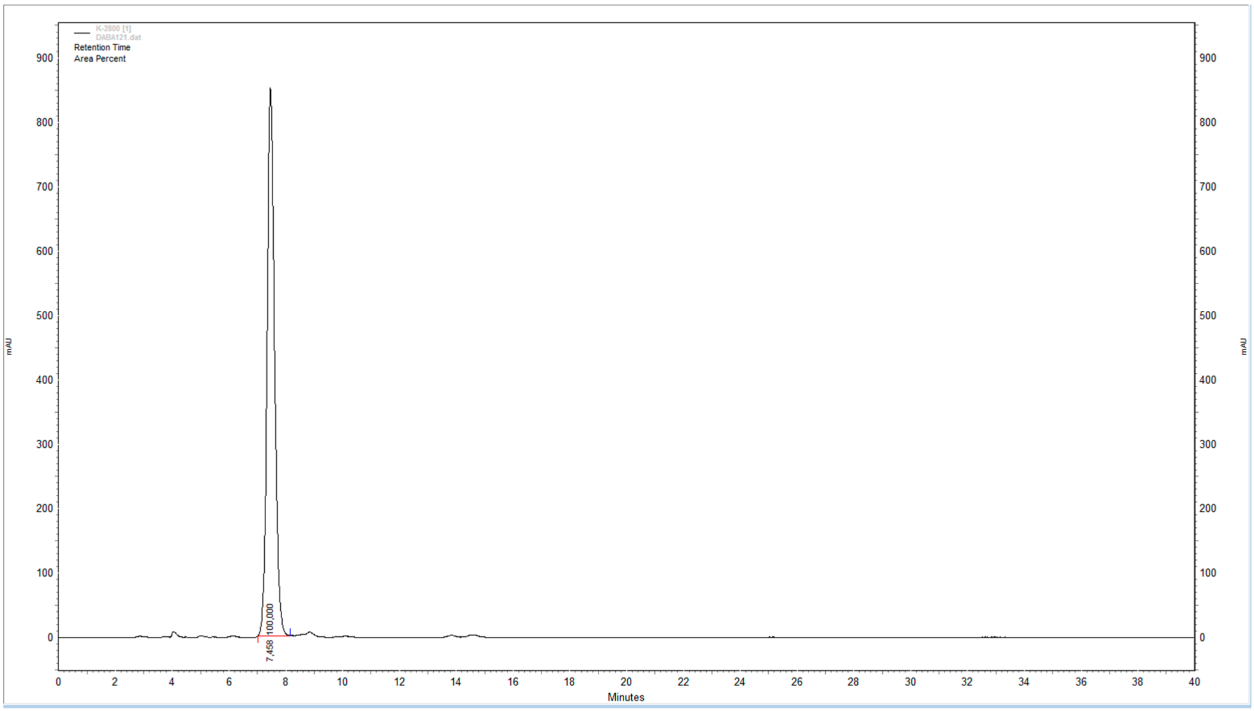

Figure S7. $^{11}B\{^1H\}$ **NMR spectrum of** $[B_{12}H_{11}NHC(NH(CH_2)_3CH(NH_3)COOH)CH_3]*3H_2O$ **(7)**

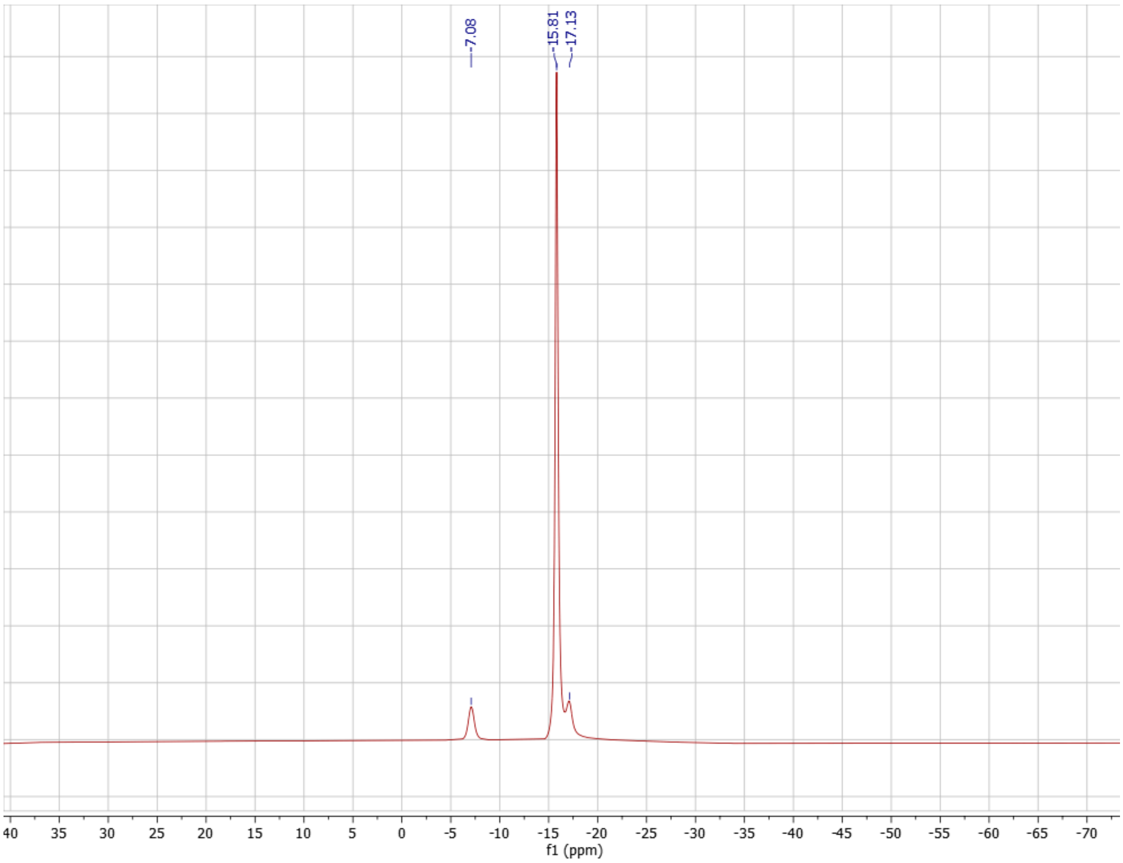

Figure S8. ^{11}B **NMR spectrum of** $[B_{12}H_{11}NHC(NH(CH_2)_3CH(NH_3)COOH)CH_3]*3H_2O$ **(7)**

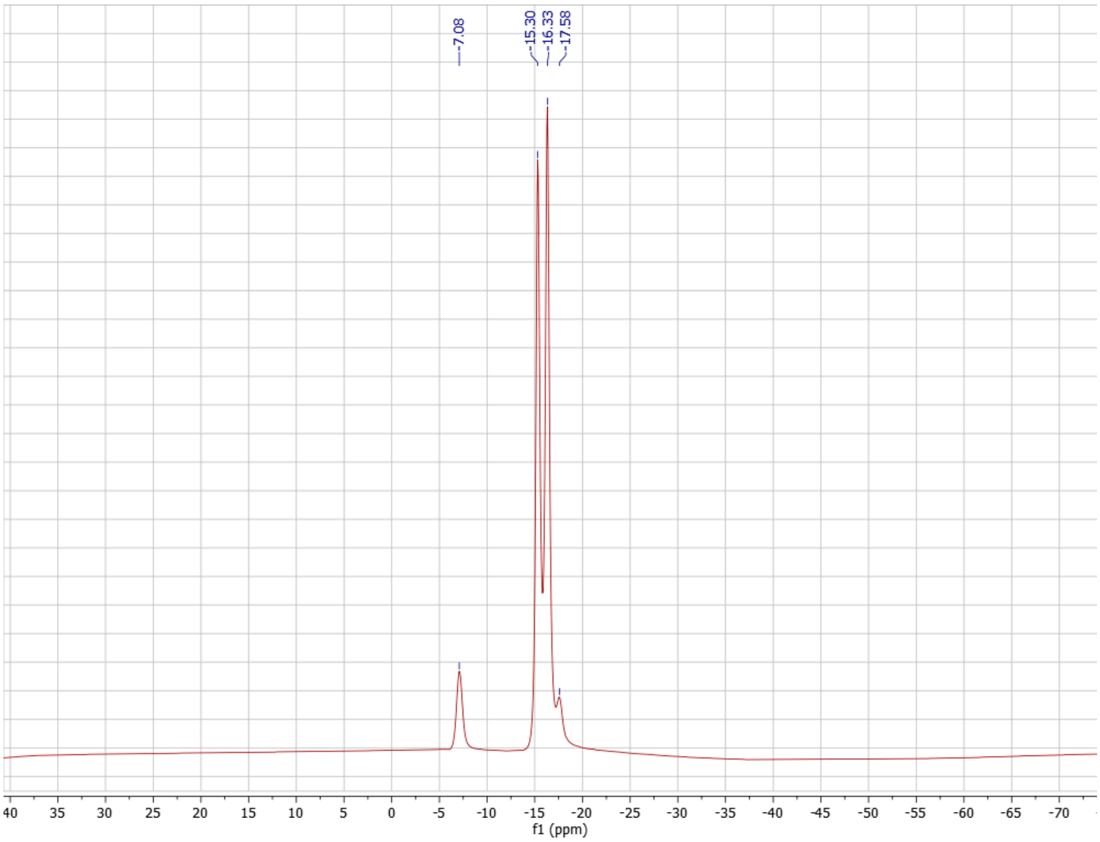

Figure S9. ^{1}H **NMR spectrum of** $[B_{12}H_{11}NHC(NH(CH_2)_3CH(NH_3)COOH)CH_3]*3H_2O$ **(7)**

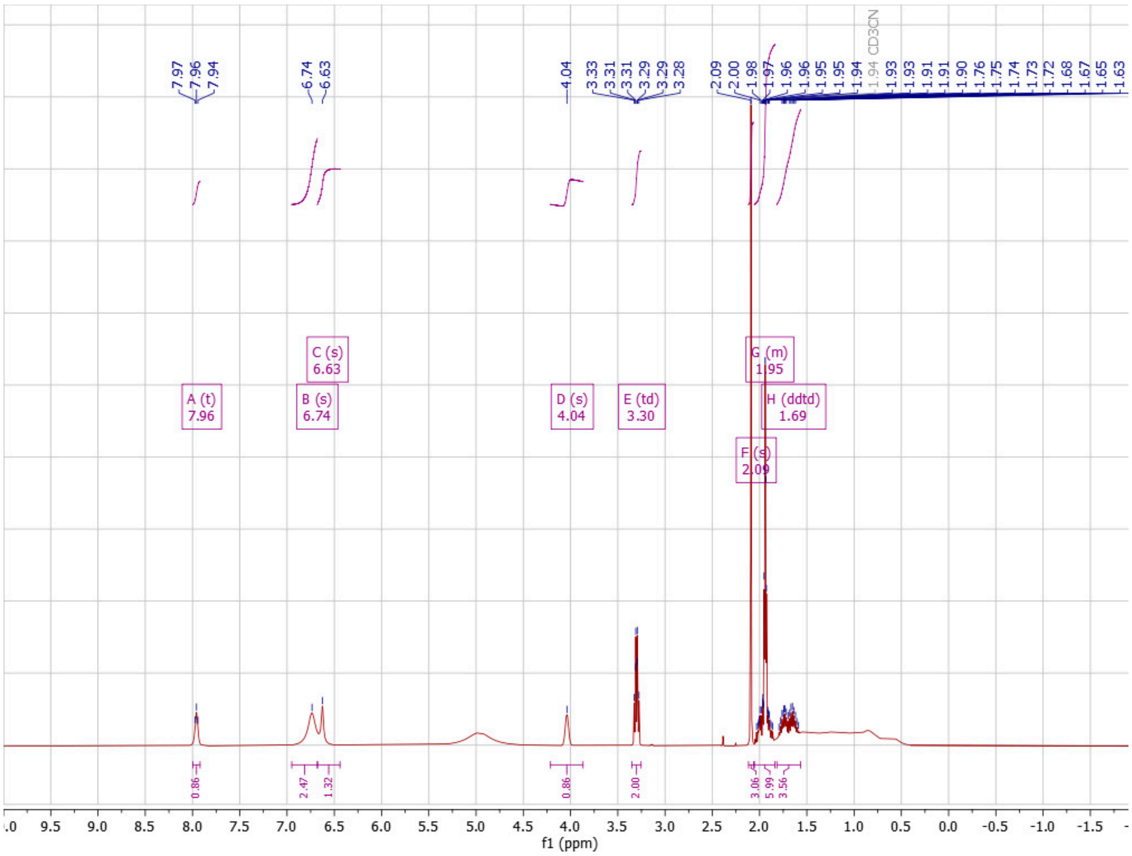

Figure S10 $^{13}C\{^{1}H\}$ **NMR spectrum of** $[B_{12}H_{11}NHC(NH(CH_2)_3CH(NH_3)COOH)CH_3]*3H_2O$ **(7)**

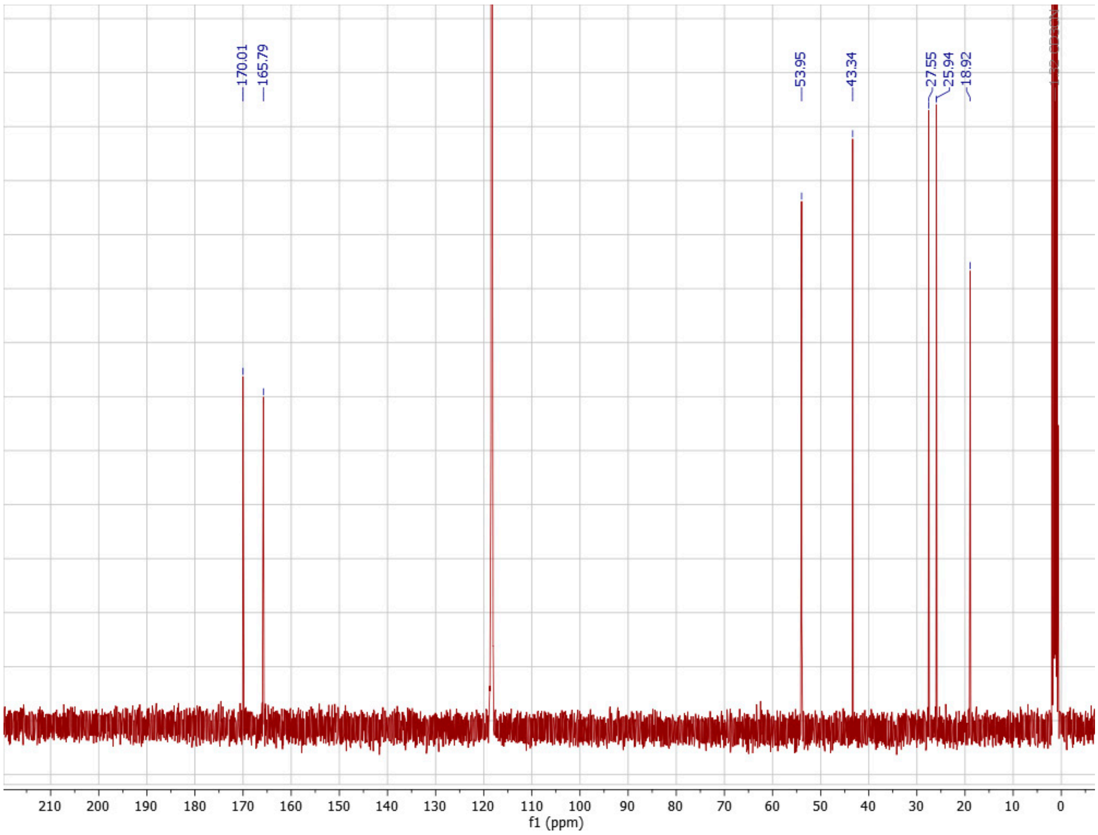

Figure S11 ESI-MS spectrum of $[B_{12}H_{11}NHC(NH(CH_2)_3CH(NH_3)COOH)CH_3]*3H_2O$ (7)

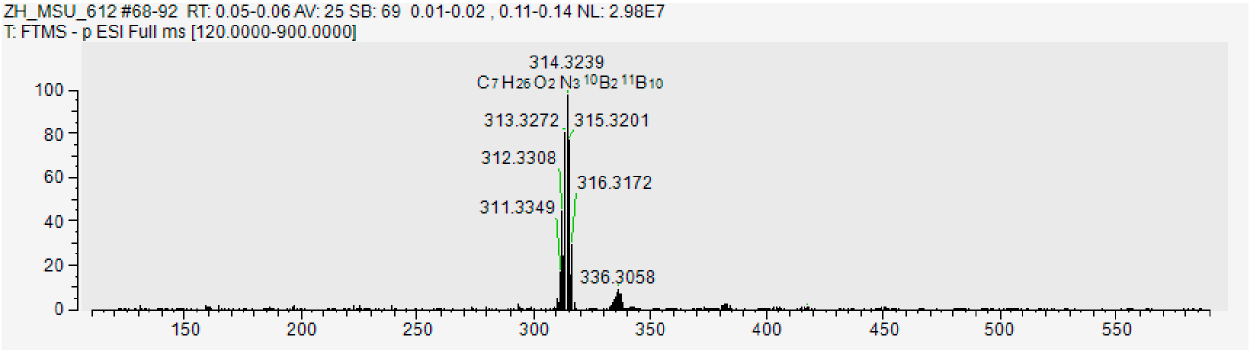

Figure S12 HPLC trace of $[B_{12}H_{11}NHC(NH(CH_2)_3CH(NH_3)COOH)CH_3]*3H_2O$ (7)

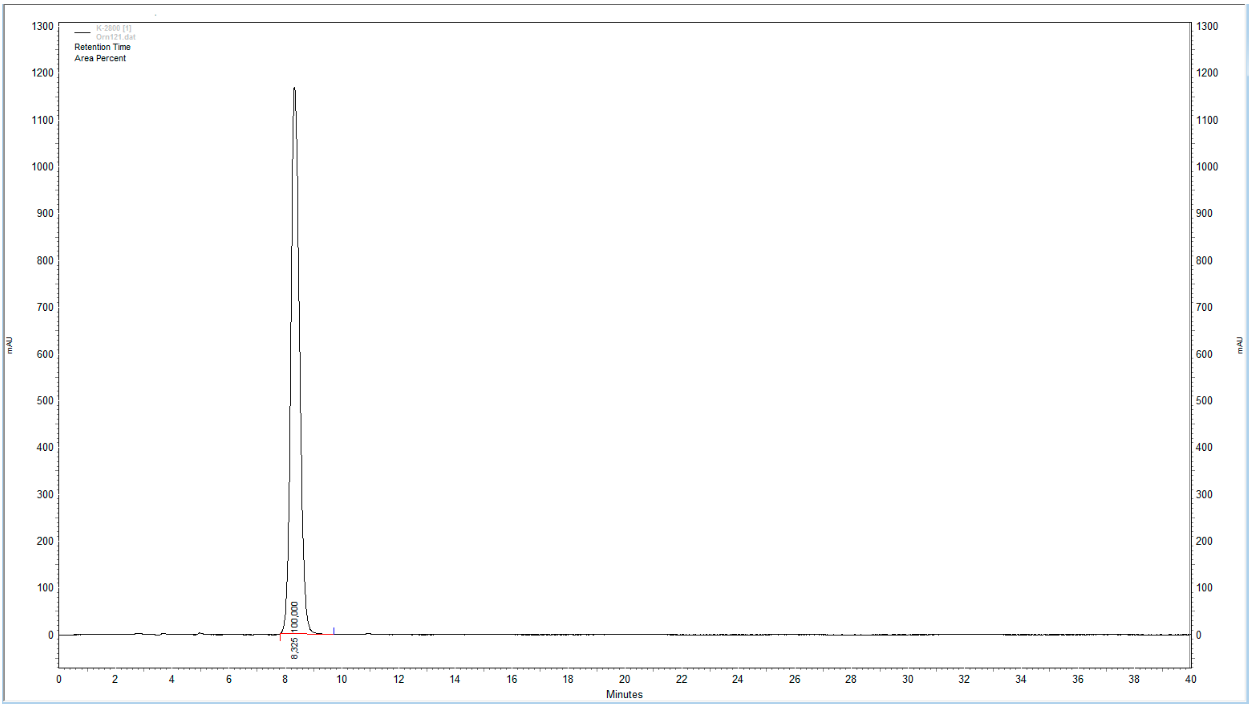

Figure S13 $^{11}B\{^1H\}$ NMR spectrum of $[B_{12}H_{11}NHC(NH(CH_2)_4CH(NH_3)COOH)CH_3]*3H_2O$ (8)

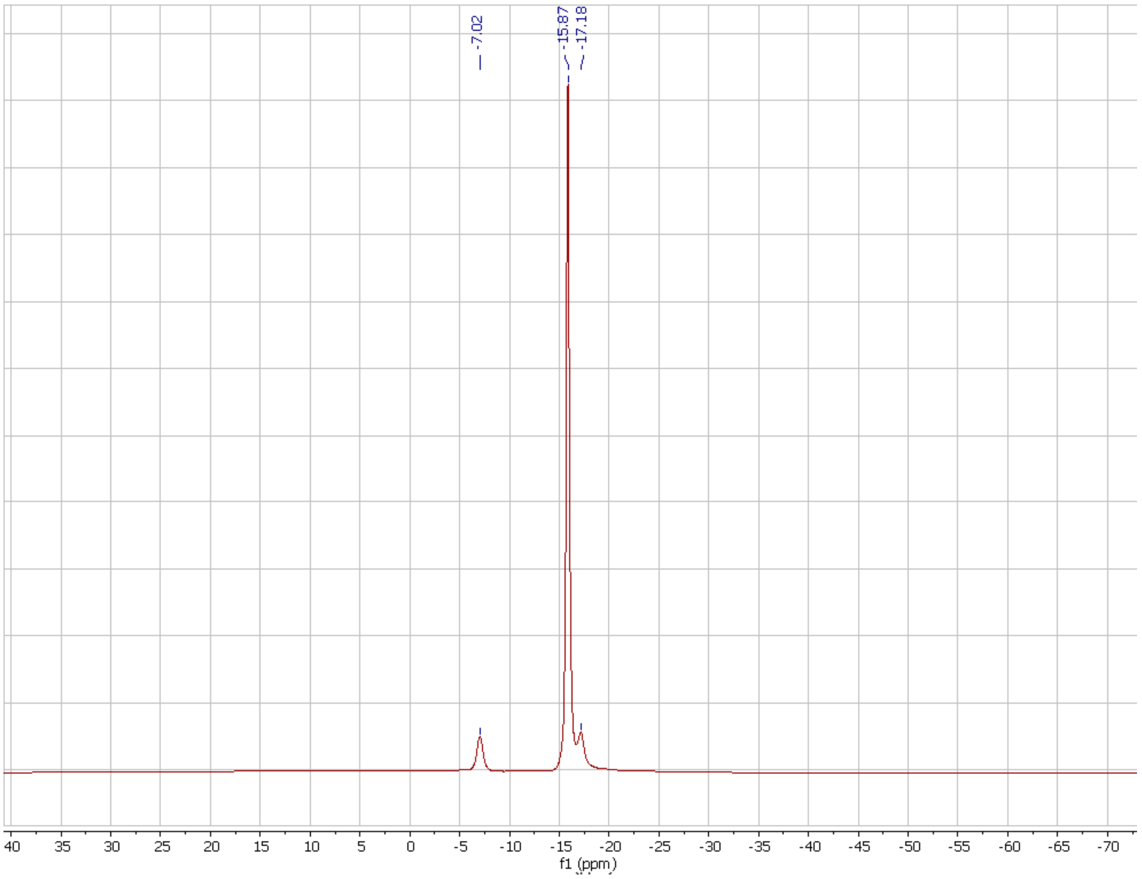

Figure S14 ^{11}B NMR spectrum of $[B_{12}H_{11}NHC(NH(CH_2)_4CH(NH_3)COOH)CH_3]*3H_2O$ (8)

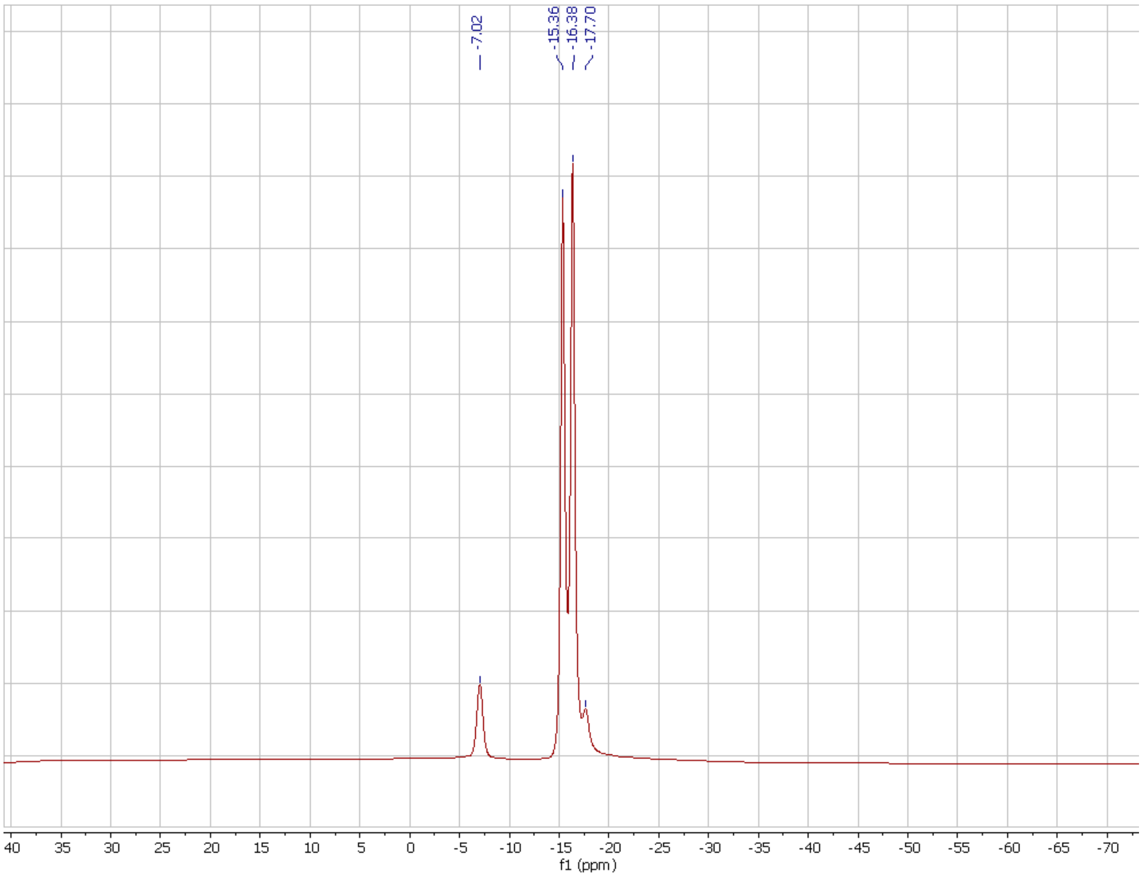

Figure S15 ^{1}H NMR spectrum of $[B_{12}H_{11}NHC(NH(CH_2)_4CH(NH_3)COOH)CH_3]*3H_2O$ (8)

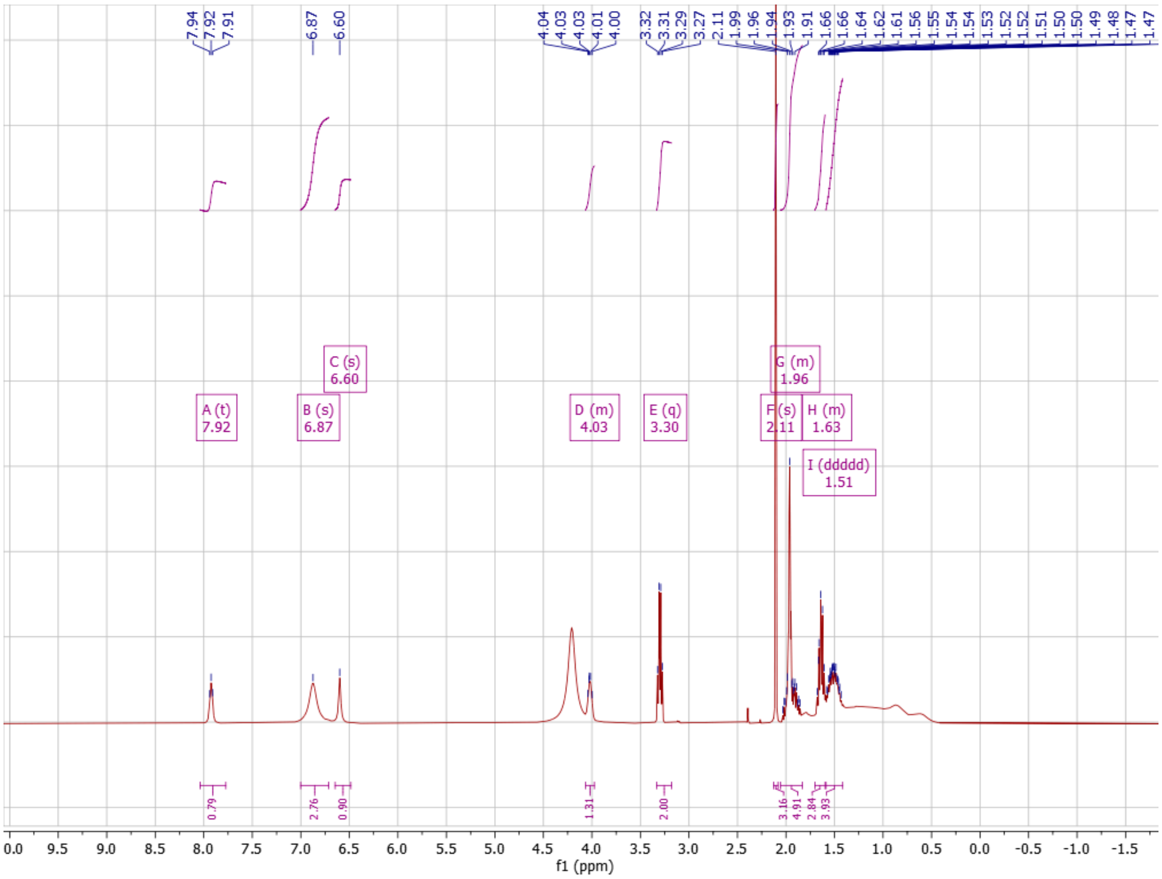

Figure S16 $^{13}C\{^{1}H\}$ NMR spectrum of $[B_{12}H_{11}NHC(NH(CH_2)_4CH(NH_3)COOH)CH_3]*3H_2O$ (8)

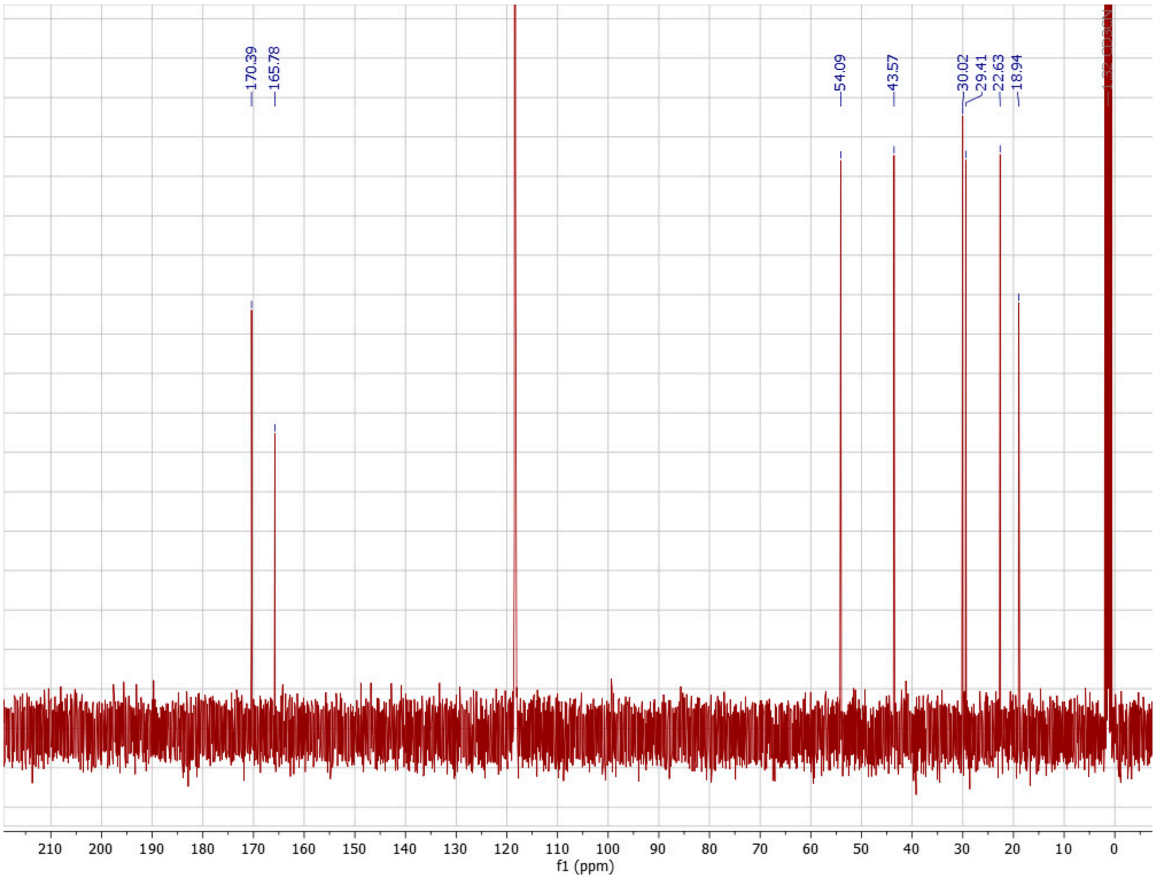

Figure S17 ESI-MS spectrum of $[B_{12}H_{11}NHC(NH(CH_2)_4CH(NH_3)COOH)CH_3]$*$3H_2O$ (8)

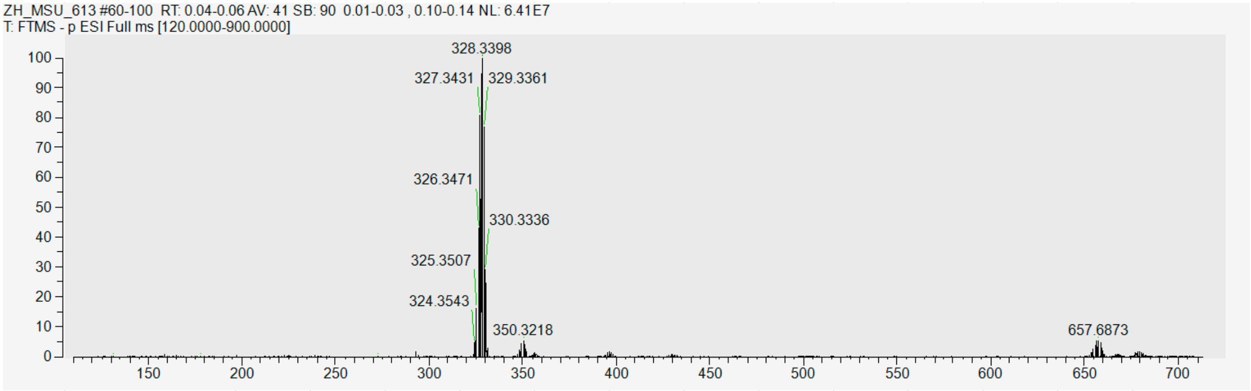

Figure S18 HPLC trace of $[B_{12}H_{11}NHC(NH(CH_2)_4CH(NH_3)COOH)CH_3]$*$3H_2O$ (8)

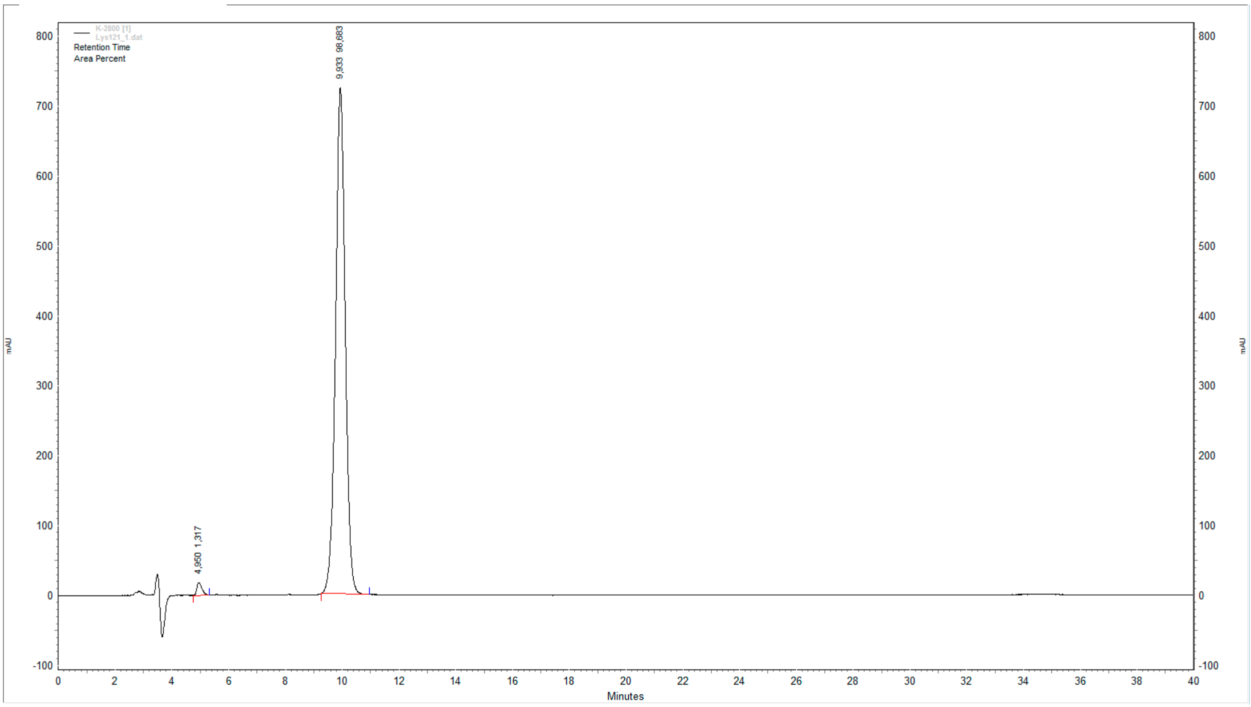

Table S1. Crystal data and structure refinement for (6) and (7).

Identification code	6	7
Empirical formula	$C_6H_{31}B_{12}N_3O_5$	$C_{13}H_{36}B_{12}N_6O_2$
Formula weight	355.06	438.20
Temperature, K	100.00	100.00
Crystal system	monoclinic	monoclinic
Space group	$P2_1$	$P2_1$
a, Å	8.7039(3)	10.1504(6)
b, Å	7.4599(3)	9.8851(6)
c, Å	15.0553(7)	12.5694(8)
α, °	90	90
β, °	103.102(3)	91.566(2)
γ, °	90	90
Volume, $Å^3$	952.10(7)	1260.71(13)
Z	2	2
ρ_{calc}g, cm^3	1.238	1.154
μ, mm^{-1}	0.651	0.069
F(000)	376.0	464.0
Radiation	CuKα (λ = 1.54178)	MoKα (λ = 0.71073)
2Θ range, °	6.028 to 145.826	3.242 to 51.986
Reflections collected	11070	14320
Independent reflections	3500 [R_{int} = 0.0737]	4962 [R_{int} = 0.0393]
GooF on F^2	1.133	1.055
Final R indexes [I>=2σ (I)]	R_1 = 0.0752, wR_2 = 0.2038	R_1 = 0.0316, wR_2 = 0.0782
Final R indexes [all data]	R_1 = 0.0863, wR_2 = 0.2188	R_1 = 0.0342, wR_2 = 0.0806

Table S2. Boron content in organs and tissues of C57Bl/6 mice with subcutaneous B16F10 melanoma after intravenous injection of $[B_{12}H_{11}NHC(NH(CH_2)_2CH(NH_3)COOH)CH_3]*3H_2O$ (6)

Time after injection, min	Blood, µg/g	Tumor, µg/g	Muscle, µg/g	Liver, µg/g	Kidneys, µg/g
15	41±8	17±2	6,3±1,4	88±8	275±57
30	14±3	11±4	2,1±0,7	95±14	95±17
45	9±3	8±2	1,3±0,5	33±9	59±13
60	7±2	6,1±1,8	1,6±0,5	32±8	52±16
90	3,1±0,8	3,1±0,6	1,2±0,4	10±2	34±10
120	2,1±0,5	2,5±0,7	0,85±0,18	3,5±0,8	20±4

Table S3. Boron content in organs and tissues of C57Bl/6 mice with subcutaneous B16F10 melanoma after intravenous injection of boronophenylalanine

Time after injection, min	Blood, µg/g	Tumor, µg/g	Muscle, µg/g	Liver, µg/g	Kidneys, µg/g
15	17±1	33±3	17±3	202±35	343±19
30	13±1	37±3	13±1	29±2	69±6
45	11±1	30±4	14±3	9±2	39±6
60	8,8±0,3	26±2	13±1	7,4±0,4	29±3
90	7,7±0,6	17±3	12±3	6,4±0,5	26±2
120	6,2±0,9	18±2	12±2	4,8±0,5	19±8

Table S4. Tumor-to-Normal Muscle Tissue Boron Content Ratio (T/N ratio) in C57Bl/6 Mice with Subcutaneous B16F10 Melanoma after Intravenous Injection of $[B_{12}H_{11}NHC(NH(CH_2)_2CH(NH_3)COOH)CH_3]*3H_2O$ (6) and Boronophenylalanine

Time after injection, min	T/N gamma-DABA	T/N BPA
15	2,7±0,7	2,0±0,4
30	5,3±2,4	2,9±0,3
45	6,2±2,8	2,1±0,5
60	3,9±1,8	1,9±0,3
90	2,6±1,1	1,4±0,4
120	2,9±1,1	1,5±0,3

Table S5. Boron Content in Organs and Tissues of Balb/C Mice with Subcutaneous 4T1 Adenocarcinoma after Intravenous Injection of $[B_{12}H_{11}NHC(NH(CH_2)_2CH(NH_3)COOH)CH_3]*3H_2O$ (6)

Time after injection, min	Blood, μg/g	Tumor, μg/g	Muscle, μg/g
15	30±7	13±3	4,4±1,0
30	14±3	5,4±1,3	2,9±0,8
60	8±2	3,4±1,2	2,1±0,7
90	4,8±1,6	2,6±1,0	2,0±0,6
120	3,7±1,2	1,5±0,4	2,2±0,6

Table S6. Boron Content in Organs and Tissues of Balb/C Mice with Subcutaneous 4T1 Adenocarcinoma after Intravenous Injection of Boronophenylalanine

Time after injection, min	Blood, μg/g	Tumor, μg/g	Muscle, μg/g
15	17±1	12±1	13,8±0,8
30	12±1	16±1	12,3±0,8
60	11±1	17±1	8,6±0,4
90	8,1±1,4	17±3	8,1±0,8
120	6,2±0,8	15±3	8,4±0,9

Table S7. Tumor-to-Normal Muscle Tissue Boron Content Ratio (T/N ratio) in Balb/C Mice with Subcutaneous 4T1 Adenocarcinoma after Intravenous Injection of (6) and Boronophenylalanine

Time after injection, min	T/N (6)	T/N BPA
15	3,0±0,9	0,9±0,1
30	1,9±0,7	1,3±0,1
60	1,6±0,8	2,0±0,2
90	1,3±0,7	2,0±0,4
120	0,7±0,3	1,8±0,4
